# Molecular Mechanisms for Herpes Simplex Virus Type 1 Pathogenesis in Alzheimer’s Disease

**DOI:** 10.3389/fnagi.2018.00048

**Published:** 2018-03-06

**Authors:** Steven A. Harris, Elizabeth A. Harris

**Affiliations:** ^1^St. Vincent Medical Group, Northside Internal Medicine, Indianapolis, IN, United States; ^2^Department of Neurology, University of Chicago Medical Center, Chicago, IL, United States

**Keywords:** Alzheimer’s disease, amyloid beta, dementia, herpes simplex virus, neurodegeneration, neuroinflammation, pathogen, tau

## Abstract

This review focuses on research in the areas of epidemiology, neuropathology, molecular biology and genetics that implicates herpes simplex virus type 1 (HSV-1) as a causative agent in the pathogenesis of sporadic Alzheimer’s disease (AD). Molecular mechanisms whereby HSV-1 induces AD-related pathophysiology and pathology, including neuronal production and accumulation of amyloid beta (Aβ), hyperphosphorylation of tau proteins, dysregulation of calcium homeostasis, and impaired autophagy, are discussed. HSV-1 causes additional AD pathologies through mechanisms that promote neuroinflammation, oxidative stress, mitochondrial damage, synaptic dysfunction, and neuronal apoptosis. The AD susceptibility genes apolipoprotein E (*APOE*), phosphatidylinositol binding clathrin assembly protein (*PICALM*), complement receptor 1 (*CR1*) and clusterin (*CLU*) are involved in the HSV lifecycle. Polymorphisms in these genes may affect brain susceptibility to HSV-1 infection. *APOE*, for example, influences susceptibility to certain viral infections, HSV-1 viral load in the brain, and the innate immune response. The AD susceptibility gene cholesterol 25-hydroxylase (*CH25H*) is upregulated in the AD brain and is involved in the antiviral immune response. HSV-1 interacts with additional genes to affect cognition-related pathways and key enzymes involved in Aβ production, Aβ clearance, and hyperphosphorylation of tau proteins. Aβ itself functions as an antimicrobial peptide (AMP) against various pathogens including HSV-1. Evidence is presented supporting the hypothesis that Aβ is produced as an AMP in response to HSV-1 and other brain infections, leading to Aβ deposition and plaque formation in AD. Epidemiologic studies associating HSV-1 infection with AD and cognitive impairment are discussed. Studies are reviewed supporting subclinical chronic reactivation of latent HSV-1 in the brain as significant in the pathogenesis of AD. Finally, the rationale for and importance of clinical trials treating HSV-1-infected MCI and AD patients with antiviral medication is discussed.

## Introduction

Alzheimer’s disease (AD) is an inflammatory neurodegenerative disease characterized by progressive decline in cognitive abilities, behavioral abnormalities, and the loss of ability to function at work or in activities of daily living. Cognitive impairment may involve deficits in short term memory, language, visuospatial tasks, and/or executive function (McKhann et al., 2011). AD is the leading cause of dementia. It is estimated that 47 million people worldwide have dementia, with the prevalence expected to rise significantly as the population ages (Prince et al., 2016). Early-onset AD (EOAD) presents in younger patients prior to age 60 or 65 years and comprises approximately 1%–6% of all AD cases. About 60% of these patients are classified as familial EOAD, having multiple relatives diagnosed with the disease. Approximately 13% of these cases show an autosomal dominant pattern of inheritance (Alonso Vilatela et al., 2012). The autosomal dominant form of EOAD is caused by overproduction of amyloid beta (Aβ) due to mutations in one of three genes: *APP*, *PSEN1* or *PSEN2*. *APP* encodes for amyloid precursor protein while the PSEN genes encode presenilins I and II respectively (Naj and Schellenberg, 2017). Sporadic or late-onset AD (LOAD) accounts for the majority of AD cases (approximately 95%), and usually occurs after the age of 60–65 years. LOAD appears to have a multifactorial etiology involving complex interactions between environmental factors and multiple susceptibility genes, including the ε4 allele of the apolipoprotein E (*APOE*) gene (Alonso Vilatela et al., 2012; Naj and Schellenberg, 2017). Factors associated with increased risk of AD include age, cerebrovascular disease, stroke, diabetes, dyslipidemia, head injury, hypertension, smoking and obesity (Mayeux and Stern, 2012). A significant body of evidence also implicates pathogen involvement in sporadic AD (Balin et al., 2008; Miklossy, 2011a; Harris and Harris, 2015; Itzhaki et al., 2016).

The AD pathogen hypothesis states that pathogens are causative factors in the development of sporadic or LOAD. Pathogens interact with genetic and environmental factors to initiate accumulation and/or formation of Aβ, hyperphosphorylation of tau proteins, and inflammation in the brain. This leads to neuronal cell dysfunction, neurodegeneration and dementia (Harris and Harris, 2015). The hypothesis is supported by research data implicating brain infections by herpes simplex virus type 1 (HSV-1; Itzhaki et al., 1997; Itzhaki, 2014, 2016; Steel and Eslick, 2015), *Chlamydophila pneumoniae* (Balin et al., 1998, 2008; Gérard et al., 2006), *Borrelia burgdorferi* and other spirochetes (Miklossy, 2011a,b), and fungi (Alonso et al., 2014a,b, 2015; Pisa et al., 2015a,b) in the pathogenesis of AD. These pathogens are prevalent in AD brains and can evade the host immune system forming latent or chronic infections. Neuronal cell infection by HSV-1, *C. pneumoniae* and *Borrelia burgdorferi* induce amyloid beta (Aβ) deposition *in vitro* and/or in mouse brain models (Little et al., 2004; Miklossy et al., 2006a; Wozniak et al., 2007). Neuronal cell infection by either HSV-1 or *B. burgdorferi* results in hyperphosphorylation of tau proteins (Miklossy et al., 2006a; Wozniak et al., 2009a). Pathogens can directly and indirectly induce neuroinflammation as well as neuronal dysfunction and death, which are important aspects of AD pathophysiology (Athmanathan et al., 2001; Boelen et al., 2007; Balin et al., 2008; Zambrano et al., 2008; Miklossy, 2011a; Harris and Harris, 2015). Additional microbes associated with AD include *Helicobacter pylori* (Kountouras et al., 2009; Roubaud Baudron et al., 2013; Wang X. L. et al., 2014), cytomegalovirus (CMV; Strandberg et al., 2003; Lurain et al., 2013), human herpes virus 6 (Carbone et al., 2014), Epstein-Barr virus (Carbone et al., 2014), and the oral pathogens *P. gingivalis* and *T. forsythia* (Kamer et al., 2009).

This review focuses on the involvement of HSV-1 as a causative cofactor in sporadic AD. HSV-1 is prevalent in aged normal and AD brains (Jamieson et al., 1991, 1992). When present in the brains of *APOE*-ε4 allele carriers, the virus is associated with increased risk of AD (Itabashi et al., 1997; Itzhaki et al., 1997; Lin et al., 1998). Evidence is presented involving molecular mechanisms whereby HSV-1infection promotes AD pathogenesis.

## Pathological Hallmarks of Alzheimer’s Disease

The hallmark pathological features of the AD brain include senile plaques and neurofibrillary tangles (NFTs). Senile plaques are extracellular and contain Aβ which is formed by cleavage of the integral neuronal cell membrane glycoprotein amyloid-β precursor protein (AβPP) by the enzymes β-secretase and γ-secretase. Within this amyoidogenic pathway, the extracellular domain of AβPP is cleaved by β-secretase, which releases the N-terminal soluble fragment sAPPβ into the extracellular space. The enzyme γ-secretase then cleaves the intramembranous C-terminal fragment (βCTF), also known as C99, to form Aβ and APP intracellular domain (AICD). Within the non-amyoidogenic pathway, Aβ is not formed because AβPP is cleaved by α-secretase, releasing a soluble protein known as sAPPα into the extracellular space. The remaining intramembranous C-terminal fragment C83 is cleaved by γ-secretase to form P3 and AICD (Stanga et al., 2010; Cárdenas-Aguayo et al., 2014). The two main isoforms of amyloid beta are Aβ_1–42_ and Aβ_1–40._ Lower levels of Aβ in the brain appear to be neurotrophic, supporting various homeostatic processes including neurogenesis, synaptic plasticity, antioxidant activity, calcium homeostasis and redox sequestration of metal ions (Cárdenas-Aguayo et al., 2014). Increased Aβ production and/or decreased clearance results in Aβ accumulation. Elevated levels of Aβ_1–42_ isoforms can aggregate to form insoluble oligomers and fibrillary configurations leading to the formation of senile plaques (De-Paula et al., 2012).

NFTs are located within neurons and are composed of abnormally hyperphosphorylated tau proteins (De-Paula et al., 2012; Rajmohan and Reddy, 2017). Tau proteins contribute to microtubule assembly and stabilization, which is important for cytoskeleton structure and axonal transport of vesicular and organelle structures by motor kinesin or motor dynein (Kolarova et al., 2012). Tau proteins are also important in regulation of synaptic plasticity and synaptic function (Mondragón-Rodríguez et al., 2013). Under physiologic conditions, phosphorylation of tau proteins by kinases is balanced by dephosphorylation by phosphatases, which maintains the equilibrium required for binding of tau proteins to microtubules. Glycogen synthase kinase-3β (GSK3β), protein kinase A (PKA), cyclin-dependent kinase 5 (cdk5), and calcium/calmodulin-dependent kinase II (CaMK-II) are important enzymes that phosphorylate tau proteins (Kolarova et al., 2012). When tau proteins are hyperphosphorylated, however, conformational changes occur. This leads to formation of paired helical filaments (PHFs) and/or NFTs and associated microtubule destabilization, synaptic damage, and neurodegeneration (De-Paula et al., 2012).

Additional cellular processes implicated in AD pathogenesis include dysregulation of calcium homeostasis (Bezprozvanny and Mattson, 2008), impaired autophagy (Nixon, 2007; Boland et al., 2008; Nixon and Yang, 2011), oxidative stress (Bonda et al., 2010; Scheff et al., 2016; Tönnies and Trushina, 2017), mitochondrial dysfunction (Wang X. et al., 2014), synaptic dysfunction (Lassmann et al., 1992; Masliah et al., 2001; Reddy et al., 2005) and neuroinflammation (Wyss-Coray and Rogers, 2012). At the tissue level, pathologic findings include neuronal cell loss, cerebral atrophy, and amyloid angiopathy (Takahashi et al., 2017). At the systems level, AD is associated with damage to the blood brain barrier (BBB; Montagne et al., 2015), cerebral artery atherosclerosis, and cerebral hypoperfusion (Lathe et al., 2014).

## Neuroinflammation and Alzheimer’s Disease

As part of the innate immune system, microglia patrol the brain as resident macrophages, providing defense against pathogen invasion. Pattern recognition receptors, such as toll-like receptors (TLRs) located on microglia cell membranes, interact with pathogen associated molecular patterns (PAMPs), such as bacterial lipopolysaccharide (LPS), peptidoglycan, lipoproteins, flagellin, viral or bacterial nucleic acids leading to microglial production of proinflammatory molecules (Miklossy, 2011a). In pathologically affected regions of the AD brain, microglia upregulate cell surface receptors related to phagocytosis and other aspects of immune response. In addition to TLRs, these include major histocompatibility complex class II (MHCII) receptors, cytokine and chemokine receptors, the receptor for advanced glycation end products (RAGE), scavenger receptors and complement receptor 3 (Wyss-Coray and Rogers, 2012).

The microglial-mediated inflammation present in AD brains involves increased levels of proinflammatory cytokines such as interleukin-1 beta (IL-1β), interleukin-6 (IL-6) and tumor necrosis factor-α (TNF-α; Ho et al., 2005). Activated microglia also produce chemokines including RANTES, CXCL8, macrophage inflammatory protein 1α (MIP-1α), MIP-1β, and monocyte chemotactic protein 1 (MCP1); complement molecules such as C1q, C3, C4 and C9; and reactive oxygen species (ROS; Wyss-Coray and Rogers, 2012). Chemokines and complement factors have been found to be upregulated in AD brains (Veerhuis et al., 1996; Xia and Hyman, 1999; Lue et al., 2001). Studies support ROS as a mediator of inflammation-related neuronal damage and AD pathogenesis (Manoharan et al., 2016; Tönnies and Trushina, 2017).

Aβ and fibrillary Aβ activate microglial RAGE receptors or CD36 and scavenger receptors respectively, causing production of proinflammatory cytokines and ROS (Block et al., 2007). Upregulation of IL-1β is associated with increased levels of neuronal AβPP and the astrocyte inflammatory protein s100β in AD brain studies (Griffin et al., 1998). IL-1β drives the neuronal production of APP with subsequent increase in Aβ (Griffin, 2013). This describes a hypothesized vicious cycle of neuroinflammation with resultant neuronal dysfunction and cellular death. Release of cytosolic compounds, membrane breakdown products, and excess glutamate by injured neurons further activates microglia and accelerates the process, leading to chronic neurodegeneration (Gao and Hong, 2008; Chami and Checler, 2012; Cai et al., 2014).

Evidence involving the adaptive immune system supports the hypothesis that peripherally activated IFN-γ-producing T cells infiltrate the brain in response to an AD-related chemotactic gradient and impaired BBB. As proposed, subsequent T cell-mediated microglial activation results in Aβ production, neuroinflammation, and neurodegeneration (Lynch, 2014). Animal studies demonstrate that peripherally produced proinflammatory cytokines, including IL-1β, IL-6 and TNFα, are transported across the BBB with subsequent cytokine/brain interactions. The author suggests this as a potential mechanism of neuropathology and brain dysfunction (Banks, 2005). Peripherally injected human IL-1α has been shown to cross the BBB and induce memory impairment in mice (Banks et al., 2001).

## The HSV-1 Alzheimer’s Disease Hypothesis

In 1982 Ball (1982) proposed that latent HSV-1 in the trigeminal ganglia might reactivate and ascend through known anatomic nerve fiber connections into the limbic areas of the brain most affected by AD pathology. HSV-1 infection with subclinical chronic encephalitis was hypothesized as causative in AD (Ball, 1982). Itzhaki et al. (1997, 2008) have proposed that recurrent reactivation of latent HSV-1 in the brain results in “limited local damage” to neurons through direct and indirect toxic effects of the virus. Acute HSV-1 encephalitis (HSE) induces limbic pathology involving the hippocampus, temporal lobes and frontal lobes-the same areas affected in AD. HSE patients are known to have chronic cognitive and behavioral symptoms similar to those seen in AD (Ball, 1982; Itzhaki, 2011). Other viral diseases associated with tau pathology and neurodegeneration include the measles virus in subacute sclerosing panencephalitis (McQuaid et al., 1994) and HIV infection in HIV-associated neurocognitive disorders (HANDs; Anthony et al., 2006; Soontornniyomkij et al., 2012; Brown et al., 2014; Mocchetti et al., 2014).

Evidence from animal studies also supports HSV-1 entry into the brain through the olfactory bulb with the virus ascending along nerve pathways into limbic system structures significantly affected in AD, including the entorhinal cortex and hippocampus (McLean et al., 1993; Mori et al., 2005). HSV-1 DNA has been detected in olfactory bulb samples by PCR in the human brain (Baringer and Pisani, 1994). Olfactory receptor neurons synapse with mitral cell neurons of the olfactory bulb, which then project to the entorhinal cortex, amygdala, and hippocampus (Mori et al., 2005). The olfactory bulb and tract demonstrate neurodegenerative pathology early in AD (Kovács et al., 2001; Christen-Zaech et al., 2003), as does the entorhinal cortex (Braak et al., 1993). Clinically impaired olfactory function is associated with increased incidence of MCI and AD (Schubert et al., 2008; Roberts et al., 2016; Woodward et al., 2017). Thus, HSV-1 infection of the olfactory system and subsequent brain infection parallels early AD pathological and clinical findings.

Lathe and Haas (2017) found increased expression of host cell viral entry receptors for HSV-1 glycoproteins gD and gB within the hippocampus using gene expression profiling from the microarray based Allen Human Brain Atlas and Human Brain Transcriptome database. The hippocampus demonstrated significantly increased gene expression of host cell viral entry receptor proteins PVRL1, TNFRFS14 and MYH9 when tested across the whole human brain. The authors suggest that these findings contribute to the susceptibility of the hippocampus to HSV-1 infection.

## HSV-1 Is Prevalent in Elderly Brains and Increases the Risk of AD in *APOE-*ε4 Carriers

Latent HSV-1 was found by polymerase chain reaction (PCR) in a high proportion (70%–100%) of sporadic AD brains and normal elderly brains involving areas of brain typically affected in AD, including the hippocampus, temporal and frontal lobes (Jamieson et al., 1991). These findings have been confirmed by several studies (Jamieson et al., 1992; Bertrand et al., 1993; Itzhaki et al., 1997; Lin et al., 1998; Cheon et al., 2001). HSV-1 was absent in younger brains (Jamieson et al., 1992). Marques et al. (2001) and Hemling et al. (2003) found HSV-1 in very low percentages of brains studied. Wozniak et al. (2005) found HSV-1 immunoglobulin G (IgG) in cerebrospinal fluid from 52% of AD patients and 69% elderly controls, and noted that the difference was not statistically significant. However, this finding does indicate that HSV-1 DNA is prevalent in elderly brains as a complete functional genome and replicates in the brain (Wozniak et al., 2005). Itzhaki et al. (1997) and Lin et al. (1998) demonstrated that HSV-1 infection in postmortem elderly brains in combination with the presence of the *APOE*-ε4 allele of the *APOE* gene increases the risk of AD by a factor of 12, with the coexistence of both factors accounting for over half the AD subjects in the study. The Itzhaki et al. (1997) results were corroborated by Itabashi et al. (1997).

## HSV-1 Prevalence, Structure and Life Cycle

HSV-1 is a member of the *Herpesviridae* family of viruses. The virus is neurotropic and is highly prevalent in the adult population (Itzhaki and Wozniak, 2008). Worldwide, an estimated 3.7 billion people (67%) have HSV-1 infection (Looker et al., 2015; World Health Organization, 2017). Prevalence generally varies by country, region and subgroup and increases with age (Smith and Robinson, 2002), with several studies demonstrating 80%–95% prevalence in populations age 50 or older from different countries or regions (Shen et al., 2015; Korr et al., 2017; Marchi et al., 2017; Nasrallah et al., 2018). After initial infection, the virus establishes latency within sensory ganglia, such as the trigeminal ganglion (TG) of the peripheral nervous system (Perng and Jones, 2010). Infection is life-long as the virus evades the host immune system. Periodic episodes of viral reactivation and replication result in active lytic lesions known as herpes labialis or cold sores (Itzhaki, 2011).

**Figure 1:**
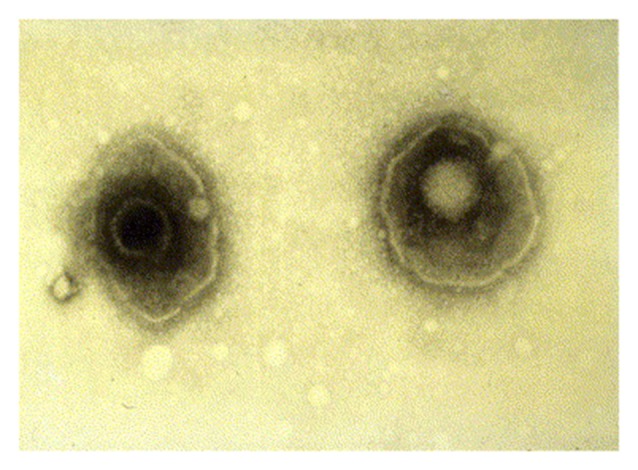
Electron microscopy image showing two herpes simplex virions. The nucleocapsid is seen in the center of each virion with surrounding tegument and viral envelope. Reprinted from Kaye and Choudhary (2006), copyright 2006, with permission from Elsevier.

HSV-1 is an enveloped virus composed of a core double stranded 152 kB DNA genome, which is surrounded by an icosahedral shaped nucleocapsid (Figure 1; Kaye and Choudhary, 2006). The tegument contains 26 viral proteins and is located between the capsid and the viral envelope. These proteins are required for the HSV viral lifecycle, including viral DNA transport to the host nucleus, viral gene transcription, and subversion of various host cellular processes. The viral envelope consists of a lipid bilayer dotted with various glycoproteins. Viral glycoproteins C (gC) and B (gB) are involved in viral attachment to the heparin sulfate proteoglycan (HSPG) receptor of the host cell. Interactions between HSV-1 glycoproteins gD, gB, and gH/gL with host cellular receptor proteins are necessary for viral entry into the host cell (Kukhanova et al., 2014). After fusion of the virus to the host cell, the tegument proteins and nucleocapsid enter the cytoplasm. A specific tegument protein shuts off host cell protein synthesis. The nucleocapsid moves from host cytoplasm to the nucleus where viral DNA is released and circularizes (Itzhaki and Wozniak, 2006).

The virus has two distinct lifecycles. During the productive lifecycle, new virions are produced leading to host cell death. During the latent lifecycle, the viral genome persists within the host cell with no virions formed. Viral genes are classified as immediate-early (α-genes), early (β-genes), or late (γ-genes; Itzhaki and Wozniak, 2006). During the productive lifecycle these genes express α proteins which regulate the viral genome, β proteins which are involved in viral DNA synthesis, and γ-proteins which are viral structural proteins (Pereira, 1996). After viral protein expression, nucleocapsids are reassembled in the host nucleus with tegument proteins attached to the outer surface of the capsid. The nucleocapsid obtains part of the host nuclear envelope upon exit from the host nucleus. Viral particles then migrate through the cytoplasm by way of the Golgi apparatus and/or endoplasmic reticulum and traverse the cell membrane to exit the cell (Itzhaki and Wozniak, 2006).

After acute infection of oral, nasal, or ocular mucosal epithelium, the virus enters the sensory neuron and moves via retrograde transport inside the axon to reach the cell body, which is located within the sensory ganglia, where the virus establishes latency (Perng and Jones, 2010; Pires de Mello et al., 2016). During latency, viral gene expression ceases except for latency-associated transcripts known as LATs, which facilitate the establishment of latency and inhibit host cell apoptosis (Perng and Jones, 2010). Immunosuppression, diseases, and other stressors can induce periodic reactivation of the virus from latency (Held and Derfuss, 2011). A cell-mediated immune response by CD8^+^ T lymphocytes, in part mediated by secretion of IFN-γ, inhibits viral reactivation from latency within infected TG (St. Leger and Hendricks, 2011; Nicoll et al., 2012). Age-related impairment of the T cell immune system is known as immunosenescence, and is associated with increased peripheral reactivation of *herpesviridae* infections including HSV-1 in the elderly (Koch et al., 2006; Stowe et al., 2007, 2012). Sawtell (1998) reviews evidence which suggests reactivation of HSV-1 occurs in only a small number of latently infected neurons during a reactivation event. The author cites animal studies demonstrating that during reactivation only a few neurons within peripheral ganglia express lytic viral proteins or Infected-cell protein 0 (ICP0) RNA.

## Molecular Mechanisms: HSV-1 Induces AD Pathophysiology and Pathology

### Neuronal Cells Infected by HSV-1 Produce Aβ and Demonstrate Altered AβPP Metabolism

**Figure 2:**
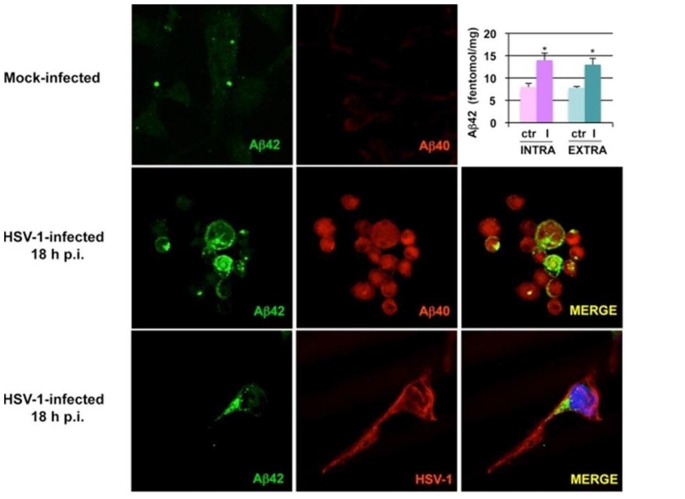
Herpes simplex virus type 1 (HSV-1) infection of neuronal cells results in accumulation of amyloid beta (Aβ). Images from confocal microscopy demonstrate human neuroblastoma cells infected by HSV-1 at 18 h post-infection. Cells shown in the middle panels were double-labeled with anti-Aβ_1–42_ and anti-Aβ_1–40_ antibodies. Cells shown in the lower panels were double-labeled with anti-Aβ_1–42_ and anti-HSV-1 antibodies. The color of the fluorescence for each primary antibody is demonstrated in the left and middle columns. Bar graph upper right shows significant increases in intracellular and secreted extracellular Aβ_1–42_ by HSV-1-infected APP695-transfected neuroblastoma cells compared to mock-infected cells by ELISA (*P < 0.05 vs. HSV-1). Figure from De Chiara et al. (2010). Reprinted under the terms of the Creative Commons Attribution License (http://creativecommons.org/licenses/by/2.0).

Human cultured neuronal cells infected with HSV-1 *in vitro* produce Aβ_1–42_ and Aβ_1–40_ with a corresponding decrease in amyloid beta precursor protein (AβPP). In addition, HSV-1-infected neuronal cells demonstrate upregulation of β-secretase and nicastrin (a protein component of the γ-secretase complex). Both enzymes are involved in processing AβPP to Aβ within the amyoidogenic pathway (Wozniak et al., 2007). Non-transgenic BALB/c mice infected with HSV1 developed brain deposition of Aβ_1–42_ detected by immunocytochemistry 5 days after intranasal inoculation (Wozniak et al., 2007). HSV-1 infection of human neuroblastoma cells and rat cortical neurons activates the host cell amyoidogenic pathway, resulting in multiple cleavages of AβPP with accumulation of intracellular and secreted extracellular Aβ_1–42_, Aβ_1–40_, and several additional neurotoxic Aβ-containing AβPP fragments. Quantitative measurements of APP 695-transfected neuroblastoma cells by ELISA demonstrated significantly increased Aβ_1–42_ levels in HSV-1-infected cells compared to mock-infected controls (Figure 2; De Chiara et al., 2010). Mechanistically, HSV-1 activates double-stranded (ds) RNA-activated protein kinase (PKR) in neuronal cells, which results in phosphorylation of eukaryotic initiation factor 2-α (eIF2-α), a GTP-binding protein involved in the initiation of protein translation. This in turn activates translation of β-secretase (Ill-Raga et al., 2011). In a squid model, GFP-labeled HSV-1 viral particles travel with AβPP, a receptor for kinesin, during fast anterograde axonal transport, mechanistically linking the virus with a key protein involved in the amyoidogenic pathway and AD (Satpute-Krishnan et al., 2003). HSV-1 affects AβPP processing within infected neuronal cells by reducing the level of AβPP and increasing the level of a 55 kDa C-terminal AβPP fragment which includes Aβ (Shipley et al., 2005). Newly synthesized HSV-1 particles co-localize and travel with AβPP inside the cytoplasm of live epithelial cells. HSV-1 interacts frequently with AβPP and interferes with AβPP transport and distribution as the virus exits the cell (Cheng et al., 2011). Thus, HSV-1 hinders AβPP transport, alters its intracellular kinetics, and upregulates its amyoidogenic processing, resulting in the production of Aβ.

### Neuronal Cell Infection by HSV-1 Results in Hyperphosphorylation of Tau Protein

**Figure 3:**
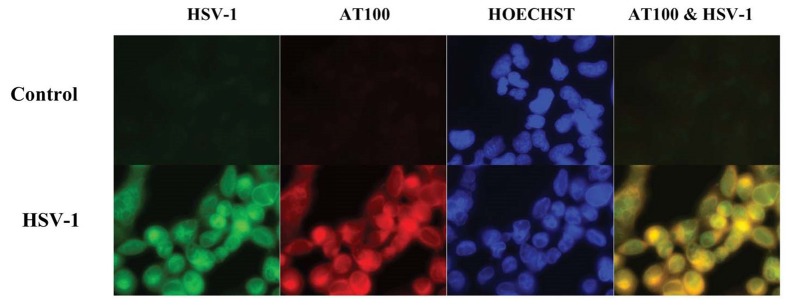
Co-localization of HSV-1 and abnormal tau phosphorylation as shown by immunofluorescence in HSV-1-infected cultured human glioblastoma cells. HSV-1-infected glioblastoma cells show strong staining for HSV-1 proteins (green) by anti-HSV-1 antibody and abnormally phosphorylated tau proteins (red) by anti-p-tau antibody AT100, with co-localization within cells seen on far right slide. Abnormal tau phosphorylation occurred in HSV-1-infected cells and not in bystander cells. DNA is stained blue with Hoechst solution. Reprinted from Wozniak et al. (2009a), copyright 2009, with permission from IOS Press and Ruth Itzhaki. The publication is available at IOS Press through http://dx.doi.org/10.3233/JAD-2009-0963.

**Figure 4:**
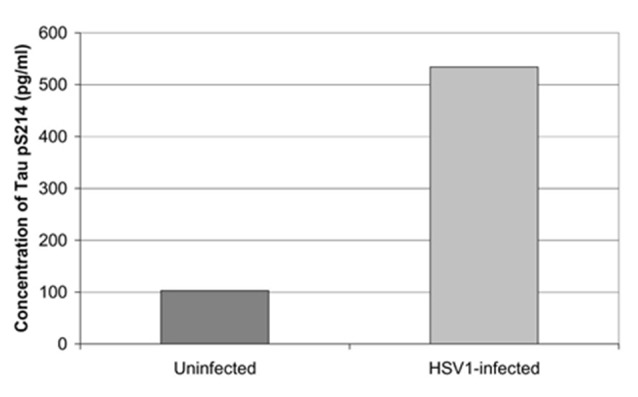
Quantification of abnormal tau phosphorylation in HSV-1-infected and uninfected cultured human neuroblastoma cells using the enzyme-linked immunosorbent assay. HSV-1-infected cells significantly hyperphosphylated tau protein at serine 214 compared to uninfected cells (*p* < 0.01). Reprinted from Wozniak et al. (2009a), copyright 2009, with permission from IOS Press and Ruth Itzhaki.The publication is available at IOS Press through http://dx.doi.org/10.3233/JAD-2009-0963.

Cultured human neuronal cells infected by HSV-1 hyperphosphorylate tau protein significantly more than uninfected cells—by a factor of four (Figures 3, 4). HSV-1 upregulates GSK3β and PKA, which are enzymes involved in phosphorylation of tau proteins (Wozniak et al., 2009a). Likewise, neuroblastoma cells infected by HSV-1 develop increased levels of hyperphosphorylated tau proteins within their nuclei (Alvarez et al., 2012). Cultured murine neuronal cells infected by HSV-1 undergo tau hyperphosphorylation and neurodegenerative changes, including alterations in microtubule dynamics, damage to the neuronal cytoskeleton, and neuronal loss. These effects are not seen in neurons pretreated with the antiviral medication acyclovir (Zambrano et al., 2008). The capability of HSV-1 to induce hyperphosphorylation of tau proteins and neurodegeneration demonstrates another mechanistic link between HSV-1 and AD pathogenesis.

### HSV-1 Induces Intracellular Aβ Accumulation by Dysregulating Calcium Homeostasis

Dysregulation of calcium homeostasis has been implicated in AD pathophysiology. Influx of calcium with resultant elevated intracellular calcium levels occurs in neuronal cells exposed to toxic Aβ oligomers and is associated with excitotoxicity and neuronal apoptosis in cultured cell and animal models (Bezprozvanny and Mattson, 2008). Elevated levels of calcium have been shown in AD triple transgenic mouse neurons, which accumulate Aβ (Lopez et al., 2008). Altered expression of neuronal calcium signaling genes has been shown in human postmortem AD brains by microarray analysis (Emilsson et al., 2006).

Piacentini et al. (2011) demonstrate that infection of rat cortical neurons with HSV-1 results in hyperexcitability and depolarization of the cell membrane due to alterations in sodium and potassium currents. This in turn leads to dysregulation of cellular calcium influx through voltage-gated calcium channels. Elevated intracellular calcium levels and calcium-dependent phosphorylation of AβPP result in elevated levels of Aβ in HSV-1-infected cells. This study shows yet another parallel between HSV-1 and AD-related pathophysiology.

### HSV-1 Impairs Autophagy in Neuronal Cells

Autophagy, or degradation of intracellular proteins and organelles in lysosome/vacuole compartments, allows for these components to be recycled (Nixon and Yang, 2011). Impaired autophagy has been demonstrated in the AD brain. Electron microscopy shows abnormal, swollen neurites containing many autophagic vacuoles, which are not found in normal brains (Boland et al., 2008). The endosomal-lysosomal pathway is important in AβPP processing. Studies suggest that increased initiation of autophagy and decreased clearance of Aβ-containing autophagic vacuoles may contribute to Aβ accumulation in the AD brain (Nixon, 2007).

Xenophagy is the autophagic degradation of intracellular pathogens including viruses, and is an important part of host defense (Alexander and Leib, 2008). The lysosomal breakdown of pathogenic components within autophagosomes and subsequent presentation of pathogenic ligands and antigens activates the host’s innate and adaptive immune systems (Orvedahl and Levine, 2008). HSV-1 viral particles have been demonstrated within lysosomes of infected human fibroblast cells (Smith and de Harven, 1978). HSV-1 degradation also takes place in autophagosomes of infected murine-embryonic fibroblast cells (Tallóczy et al., 2006). Xenophagy of HSV-1 is dependent on activation of a double stranded RNA-dependent protein kinase R (PKR) and eukaryotic initiating factor-2-α (eIF2α) pathway (Tallóczy et al., 2002, 2006). HSV-1 neurovirulence factor infected cell protein 34.5 (ICP 34.5) and viral protein Us11 function to subvert the autophagic response to viral infection. HSV-1 Us11 blocks PKR phosphorylation of eIF2α. Viral ICP 34.5 recruits host phosphatase PPP1CA which dephosphorylates eIF2α. Both of these actions inhibit autophagic degradation of HSV-1 proteins (O’Connell and Liang, 2016). HSV-1 ICP 34.5 also inhibits autophagy by binding to Beclin 1, which is an essential autophagy protein (Orvedahl et al., 2007; Wilcox and Longnecker, 2016).

**Figure 5:**
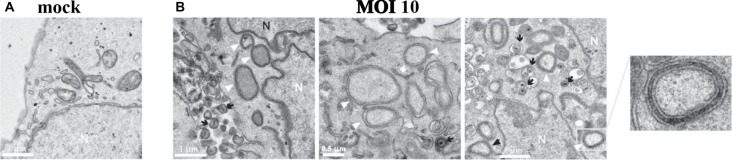
HSV-1 infection of human neuroblastoma cells leads to accumulation of intracellular autophagosomes. **(A)** Electron micrograph of mock-infected neuroblastoma cells. **(B)** Electron micrographs of HSV-1-infected neuroblastoma cells at a multiplicity of infection (MOI) of 10 plaque forming units per cell (pfu/cell) for 18 h. Micrographs show accumulation of autophagosomes (white arrowheads) induced by HSV-1. White arrows show phagophores. Numerous cytoplasmic viral vesicles and free cytoplasmic virions are visualized (black arrows). Black arrowheads point to four-layered membrane vesicles. Note the enlarged boxed area (far right) showing a four-layered membrane vesicle. N labels the nucleus. Scale bars = 0.5 or 1 μm. Reprinted from Santana et al. (2012a), copyright 2012, with permission from IOS Press and Jesus Aldudo. The publication is available at IOS Press through http://dx.doi.org/10.3233/JAD-2012-112000.

HSV-1 infection of human neuroblastoma cells impairs autophagy and leads to accumulation of intracellular autophagosomes (Santana et al., 2012a; Figure 5). HSV-1 infection was also found to decrease autophagic degradation of Aβ, resulting in intracellular accumulation of Aβ in autophagic compartments within neuroblastoma cells. Autophagosomes containing Aβ in HSV-1 infected neuroblastoma cells failed to fuse with lysosomes, resulting in a significant decrease in Aβ secretion. Inhibition of the non-amyoidogenic AβPP processing pathway was noted while the amyoidogenic Aβ producing pathway remained intact (Santana et al., 2012b). This data suggests that HSV-1 inhibition of host cell autophagy and viral-induced alterations of AβPP processing results in intraneuronal Aβ accumulation (Santana et al., 2012b).

### HSV-1 Induces Neuroinflammation

HSV-1 infection of the brain or peripheral nervous system provokes an innate and adaptive immune response (Nicoll et al., 2012; Egan et al., 2013; Shives et al., 2017). Human microglia cells infected by HSV-1 increase production of pro-inflammatory cytokines IL-1β, IL-6, IL-8 and TNF-α, along with chemokines MIP-1α, CCL5 (RANTES) and CXCL10 (Lokensgard et al., 2001). Activated CD8^+^ T cells surround human and mouse HSV-1-infected TG ganglia in an attempt to control viral reactivation (Nicoll et al., 2012). HSV-1 infection of mouse trigeminal ganglia resulted in expression of MHCII antigens and cellular infiltrates containing pro-inflammatory cytokines IL-6, TNF-α and interferon-γ (IFN-γ; Shimeld et al., 1995, 1997). Indicators of neuroinflammation (phosphorylated interferon regulatory factor 3 (p-IRF3), toll-like receptor-4, and interferon α/β) and early neurodegeneration (caspase-3 cleaved tau protein (TauC3) and phosphorylated tau protein) are found in the trigeminal ganglia and cerebral cortices of mice after HSV-1 reactivation from latency (Martin et al., 2014).

Herpes simplex encephalitis in humans induces acute and subacute inflammatory responses mediated by IFN-γ and IL-6, and TNF-α respectively. During the late convalescent stage, the T cell-mediated immune markers soluble IL-2 receptor and soluble CD8 antigen may remain elevated for months to years (Aurelius et al., 1994). Higher levels of proinflammatory response to the virus are associated with greater clinical severity, extent of BBB disruption, and amount of damage seen on brain MRI (Michael et al., 2016).

### HSV-1 Induces Oxidative Stress

Oxidative stress is characterized by an imbalance in oxidant-antioxidant equilibrium, overproduction of ROS and resultant damage to cellular macromolecules (Manoharan et al., 2016; Tönnies and Trushina, 2017). Decreased intraneuronal levels of the antioxidant glutathione have been found in AD hippocampal and cortical brain samples (Limongi and Baldelli, 2016). Oxidative stress is thought to play a highly significant role in neurodegeneration. Oxidative damage to lipids, proteins, DNA and RNA within neuronal cells is found in AD brains, and occurs early in the disease (Bonda et al., 2010; Zhao and Zhao, 2013; Scheff et al., 2016). This type of stress is associated with other aspects of AD-related pathophysiology as well, including mitochondrial dysfunction, accumulation of redox metals, dysregulation of calcium homeostasis, hyperphosphorylation of tau proteins, Aβ accumulation, synaptic dysfunction, neuroinflammation and neurodegeneration (Mondragón-Rodríguez et al., 2013; Zhao and Zhao, 2013; Tönnies and Trushina, 2017).

Herpes simplex encephalitis and other CNS viral infections increase production of reactive oxygen and nitrogen species, which contributes to oxidative stress and neuronal damage in both animal models and human disease (Meyding-Lamadé et al., 1998; Valyi-Nagy and Dermody, 2005). Keratitis due to HSV-1 infection in rabbits resulted in an altered redox state with decreased corneal intracellular levels of glutathione (Nucci et al., 2000). Mouse microglial cells infected by HSV-1 produce elevated levels of ROS through viral stimulation of microglial toll-like receptor 2. The subsequent redox imbalance results in neuronal oxidative damage characterized by lipid peroxidation in murine mixed microglial-neuronal cultures (Schachtele et al., 2010). HSV-1-infected human neuronal cells exposed to experimental oxidative stress *in vitro* demonstrate decreased Aβ secretion and accumulation of Aβ intracellularly. Oxidative stress interacts with the virus to significantly enhance the effects of HSV-1-induced Aβ accumulation and impairment of autophagy (Santana et al., 2013).

### Mitochondrial Damage and Dysfunction Occurs in Cells Infected by HSV-1

Mitochondrial damage is thought to impair ATP production and increase ROS production promoting oxidative stress (Wang X. et al., 2014). Mitochondrial dysfunction occurs early in AD (Wang et al., 2009). Damaged mitochondria are seen within neurons from AD brain biopsies using electron microscopy (Hirai et al., 2001). Mitochondrial damage is present in transgenic APP and APP/PS1 mouse models (Trushina et al., 2012) and transgenic neuronal cells *in vitro* which overexpress APP (Wang et al., 2008).

HSV-1 and pseudorabies virus (PRV), another member of the *alphaherpesviridae* sub-family, alter mitochondrial morphology and interfere with axonal transport of mitochondria in rat superior cervical ganglion neurons. During PRV infection, there is reduced recruitment of the molecular motor kinesin-1 to mitochondria. This effect is mediated by glycoprotein B (gB) fusion to the neuronal cell membrane, which results in increased neuronal action potential firing rates and elevated intracellular calcium levels (Kramer and Enquist, 2012). HSV-1 infection of Vero cells depletes mitochondrial DNA and mRNA through the action of viral protein UL12.5, which suggests a direct connection between HSV-1 infection and mitochondrial dysfunction and damage (Saffran et al., 2007).

### HSV-1 Infection Leads to Synaptic Dysfunction

Synaptic dysfunction appears to be an early event in AD pathogenesis (Masliah et al., 2001). Decreased levels of synaptophysin and other synaptic proteins have been reported (Lassmann et al., 1992; Masliah et al., 2001; Reddy et al., 2005). *In vitro* studies demonstrate that elevated Aβ and phosphorylated tau levels within and outside of the synaptic cleft trigger diverse molecular mechanisms, leading to synaptic protein reduction, synaptic damage, and synaptic loss (Rajmohan and Reddy, 2017). Cyclic AMP-response element-binding protein (CREB) is a multifunctional transcription factor which plays a key role in synaptic plasticity, learning and memory (Liang et al., 2007; Sakamoto et al., 2011). CREB also regulates molecular processes related to neurodevelopment, upregulation of antioxidant genes, and neuronal survival (Sakamoto et al., 2011). Reduced CREB activity has been found in AD postmortem brain samples and AD related animal models (Yamamoto-Sasaki et al., 1999; Matsuzaki et al., 2006; Liang et al., 2007). Aβ_1–42_ interferes with activation of CREB in cultured rat cortical neurons (Tong et al., 2001), cultured rat hippocampal neurons (Vitolo et al., 2002), and long term potentiation in a mouse hippocampus model of synaptic plasticity (Puzzo et al., 2005).

HSV-1 infection affects the synapse through mechanisms which parallel AD-related pathophysiology. Cultured mouse cortical neurons infected by HSV-1 have shown decreased synaptic transmission and reduced levels of presynaptic proteins synapsin-1 and synaptophysin. Synaptic dysfunction and lower levels and/or activity of synaptic proteins were mediated through HSV-1-related activation of GSK3β and intraneuronal Aβ accumulation. HSV-1-induced calcium-dependent GSK3β activation resulted in phosphorylation of amyloid precursor protein and subsequent accumulation of Aβ within neuronal cells. The activity of CREB was inhibited during viral infection and was dependent on HSV-1-induced Aβ accumulation and GSK3β activation (Piacentini et al., 2015).

### HSV-1 Affects Neuronal Apoptosis

HSV-1 is able to block or induce neuronal apoptosis at various stages of infection (Galvan and Roizman, 1998). HSV-1 protein ICP 34.5 dephosphorylates eIF2α, which blocks the shutdown of host cell protein synthesis and prevents apoptosis (Chou et al., 1995; Itzhaki et al., 2008). HSV-1 also inhibits host cell apoptosis through mechanisms utilizing viral proteins including LAT, gJ, gD, Us3, ICP 4, ICP 24, ICP 27 and UL14 (Yu and He, 2016). On the other hand, HSV-1 infection causes neuronal apoptosis in cultured murine neuronal cells (Zambrano et al., 2008) and a murine model for HSE (Armien et al., 2010). Neuronal apoptosis has also been demonstrated in human HSE brain tissue and cultured human glioblastoma cells infected by HSV-1 (Athmanathan et al., 2001).

## HSV-1 Interactions with AD-Related Genes

### AD Susceptibility Genes Are Involved in the HSV Lifecycle

AD susceptibility genes are characterized by single nucleotide polymorphisms (SNPs), which are associated with increased risk for AD. Susceptibility genes for AD identified in genome-wide association studies (GWAS) include *APOE*, complement receptor 1 (*CR1*), clusterin (*CLU*) and phosphatidylinositol binding clathrin assembly protein (*PICALM*; Lambert et al., 2009, 2011). These genes are associated with the HSV lifecycle, with involvement in viral entry and transport within the host cell (*PICALM, CLU*), viral infectivity (*APOE4*), viral exit from the nucleus (*PICALM*), and complement system interactions and immune defense (*CLU, CR1*; Carter, 2010). The AD susceptibility gene Nectin-2 (*NC-2*), also known as poliovirus receptor-related-2 (*PVRL-2)*, expresses the adhesion molecule known as herpes virus entrance-B (HveB; Porcellini et al., 2010). HveB is a human plasma membrane glycoprotein, which functions as a viral entry receptor. HSV-1 viral envelope glycoprotein D (gD) interacts with HveB during fusion of the viral envelope to the host cell membrane (Spear, 2004). An AD-related gene signature has been hypothesized whereby interactions among a network of AD susceptibility genes influence infectivity and brain immune response, which contributes to an individual’s predisposition to HSV-1-induced AD pathology (Porcellini et al., 2010; Licastro et al., 2011).

### APOE Polymorphisms Affect Susceptibility to Viral Infections, Cerebral HSV-1 Viral Load and Immune Response

Possession of the *APOE*-ε4 allele, also known as *APOE4*, is a major genetic risk factor for sporadic AD (Castellano et al., 2011). *APOE* codes for APOE, which is a 299 amino acid glycoprotein component of lipoproteins (Mahley and Rall, 2000). In the brain, apoE is produced by microglial cells and astrocytes. Apolipoproteins perform multiple functions within the brain related to lipid transport, regulation of lipid metabolism, synaptic plasticity, cell signaling, and neuroinflammation (Holtzman et al., 2012). The apolipoprotein isoforms apoE4, apoE3, apoE2 are products of three predominant *APOE* alleles known as *APOE*-ε4, ε3 and ε2 respectively (Kuhlmann et al., 2010). Possession of human apoe4 results in the greatest amyloid accumulation in APP transgenic mouse models. Amyloid deposition, aggregation, and fibrillization in the brain is age and apoe isoform-dependent in animal studies, with the highest Aβ burden found in apoE4 > apoE3 > apoE2 transgenic mice (Bales et al., 2002).

Apolipoprotein isoforms differentially influence the susceptibility to and outcome of several viral infectious diseases (Kuhlmann et al., 2010). Possession of *APOE-ε4* is a risk factor for recurrent herpes labialis (Itzhaki et al., 1997). HSV-1 seropositive patients possessing the *APOE-ε4* allele developed symptomatic oral herpetic lesions at higher rates compared to non-*APOE-ε4* carriers with a relative risk of 4.64 (Koelle et al., 2010). HIV patients who possess the apoE4 isoform have a higher incidence of dementia and peripheral neuropathy than HIV patients who are apoE4 negative (Corder et al., 1998). HIV patients homozygous for *APOE-ε4* had more rapid disease progression and mortality than those homozygous for *APOE-ε3* (Burt et al., 2008). Interestingly, possession of *APOE-ε4* is protective against chronic hepatitis C, and lowers the risk of developing severe liver disease from the virus compared to *APOE-ε3* (Wozniak et al., 2002; Price et al., 2006; Kuhlmann et al., 2010).

HSV-1 interacts with APOE dosage and *APOE4* genotype resulting in increased HSV-1 concentration in mouse brain. Wild-type apoE +/+ mice infected peripherally with HSV-1 were found to have HSV-1 DNA brain concentrations 13.7 times higher than HSV-1 infected apoE−/− knockout mice. *APOE4* transgenic mice infected with the virus developed HSV-1 DNA brain levels 13.6 times greater than infected *APOE3* mice (Burgos et al., 2006). Another study demonstrated that age, female gender, and apoE dosage increased HSV-1 viral load in brains of infected mice (Guzman-Sanchez et al., 2012). One hypothesized mechanism for these results suggests that apoE4 competes with HSV-1 less effectively than apoE3 and apoE2 for attachment to the viral entry receptor HSPG. ApoE4 would then allow more HSV-1 virions to infect the target cell than apoE3 or apoE2 (Itzhaki and Wozniak, 2006).

Possession of the *APOE-*ε4 allele is associated with an increased innate immune response in human subjects exposed to pathogen-associated ligands. Whole blood samples from *APOE-*ε4/*APOE-*ε3 carriers exposed *ex vivo* to TLR2 or TLR4 ligands produced significantly higher levels of IL-1β, IL-6, IFN-γ and TNF-α than whole blood samples from *APOE-*ε3/*APOE-*ε3 carriers. Enhanced immune response by *APOE-*ε4/*APOE-*ε3 carriers was also seen after intravenous exposure to bacterial LPS (Gale et al., 2014). Heightened proinflammatory responses by *APOE-*ε4 carriers to brain infections could conceivably contribute to AD-related neuroinflammation.

### The AD Susceptibility Gene CH25H Is Involved in the Antiviral Immune Response

The gene cholesterol 25-hydroxylase (*CH25H*) regulates lipid metabolism and has been shown to be a susceptibility gene for sporadic AD. Specific *CH25H* haplotypes characterized by single nucleotide polymorphisms (SNPs) are associated with increased risk for sporadic AD in four ethnically-independent populations. Expression of *CH25H* is upregulated in specific AD-affected brain regions including the temporal cortex and hippocampus. Specific *CH25H* haplotypes are associated with different levels of Aβ deposition in the brain. Elderly non-demented subjects carrying *CH25Hχ*^4^ had high levels of Aβ deposits from postmortem medial temporal lobe brain samples, whereas *CH25Hχ*^2^ carriers lacked Aβ deposits (Papassotiropoulos et al., 2005).

*CH25H* is an interferon-stimulated gene involved in the host immune response against HSV-1 and other enveloped viruses. *CH25H* encodes the enzyme CH25H which oxidizes cholesterol to 25-hydroxycholesterol (25OHC). The multifunctional oxysterol 25OHC inhibits entry of enveloped viruses including HSV-1 by blocking viral fusion to the host cell (Liu et al., 2013). 25OHC functions as part of the innate immune system, with macrophage 25OHC expression induced by TLR agonists and PAMPs including LPS, poly (I:C), and lipoteichoic acid (Lathe et al., 2014). 25OHC is upregulated within mouse macrophages in response to viral infection or stimulation by interferons. 25OHC has been shown to have potent broad-spectrum antiviral activity against enveloped viruses in various host cell systems (Blanc et al., 2013; Lathe et al., 2014).

Lathe et al. (2014) reviews evidence supporting the hypothesis whereby chronic production of 25OHC by macrophages in response to viral pathogens results in elevated levels of insoluble cholesteryl esters in the brain. As proposed, excessive production of cholesteryl esters leads to fat deposition in macrophages, formation of functionally impaired foam cells, and atherosclerosis of cerebral vessels with vascular occlusion, which contributes to AD pathology. In addition, Itzhaki et al. (2016) points out that polymorphisms in *CH25H* influence both susceptibility to AD and deposition of Aβ, suggesting that Aβ induction by 25OHC may be a potential mechanistic link between host immune response to viral infection and production of Aβ in the AD brain.

### HSV-1 Interacts with Neprilysin and GSK3β Genes via APP Intracellular Domain

The enzyme neprilysin degrades Aβ in the brain and has been implicated in AD pathophysiology, with lower levels of neprilysin found in AD brains (Yasojima et al., 2001; Marr et al., 2004; Iwata et al., 2005). The enzyme GSK3β is involved in hyperphosphorylation of tau protein and overproduction of Aβ and in the AD brain (Hooper et al., 2008). Wozniak et al. (2009a) found that HSV-1 infection of neuronal cells resulted in phosphorylation of tau proteins at AD-specific sites. The virus induced increased levels of GSK3β and PKA, enzymes which phosphorylate tau proteins at these sites.

Human neuroblastoma cells and rat cortical neurons infected by HSV-1 *in vitro* were shown to activate the amyoidogenic pathway with resultant elevated levels of AICD, which localized in the nucleus of infected cells. AICD modulated neprilysin transcription by binding to the promoter region of the neprilysin genes NEP*prom1* and NEP*prom2*, resulting in a transient increase in mRNA levels with subsequent reduction of *nep* mRNA, protein and enzymatic activity. AICD also bound to the promotor region of the *gsk3β* gene, which encodes for GSK3β. GSK3β protein levels did not change significantly; however, enzyme activity appeared to be modulated by HSV-1 infection, which continued until phosphorylation inactivated GSK3β in the later stages of infection. Thus, HSV-1 infection alters the expression of neprilysin and modulates the activity of neprilysin and GSK3β—enzymes involved in Aβ production, Aβ clearance, and hyperphosphorylation of tau protein (Civitelli et al., 2015).

### HSV-1 Alters CREB, Glutamate and Voltage-Gated Ion Channel Gene Expression in Stem Cell-Derived Neuronal Cells

Abnormalities in cognition-related pathways including CREB (Teich et al., 2015), glutamate (Thomas, 1995; Lewerenz and Maher, 2015), and voltage-gated ion channels (Shah and Aizenman, 2014; Kumar P. et al., 2016) have been associated with AD-related cognitive impairment. Microarray analysis during the lytic phase of HSV-1-infected human induced pluripotent stem cell-derived glutamatergic neurons demonstrated significant changes in neuronal gene expression involving CREB and glutamate signaling. After treatment with antiviral drugs, during the quiescent phase of infection, persistent changes in voltage-gated ion channel and glutamate receptor gene expression were also noted (D’Aiuto et al., 2015).

## HSV-1 Co-Localizes with Aβ within Amyloid Plaques

**Figure 6:**
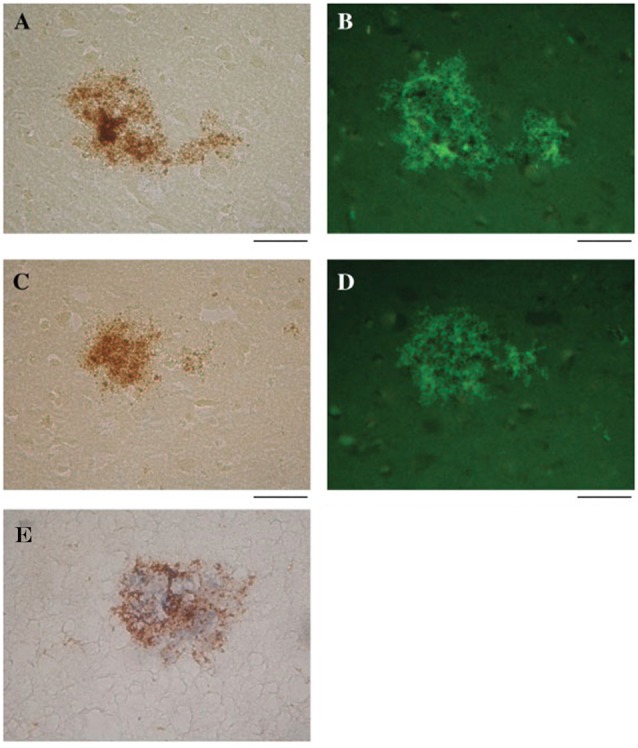
HSV-1 DNA co-localizes with amyloid plaques. *In situ* PCR was used to detect HSV-1 DNA in specimens from AD and elderly normal brains (brown staining, **A,C**, respectively). In the same tissue specimens, amyloid plaques were localized using thioflavin S (green staining, **B,D**). Note the strong co-localization of HSV-1 DNA (brown staining) and amyloid plaques stained for Aβ_1–42_ using immunohistochemistry (blue staining) **(E)**. Scale bar = 50 μm. Figure from Wozniak et al. (2009b). Reprinted with permission from John Wiley and Sons.

Wozniak et al. (2009b) found that HSV-1 DNA co-localizes with Aβ within amyloid plaques from AD and elderly normal postmortem brains. AD brains had a higher frequency of amyloid plaques and significantly more plaque-associated viral DNA compared to elderly normal brains. Thioflavin-S or Aβ_1–42_ labeled by immunohistochemistry identified amyloid plaques. In the same tissue specimens, *in situ* PCR localized HSV-1 DNA within these plaques (Figure 6; Wozniak et al., 2009b). This remarkable discovery may relate to findings by Cribbs et al. (2000), demonstrating 67% peptide homology between the HSV-1 envelope glycoprotein B (gB) and the carboxyl-terminal region of Aβ_1–42_. In addition, synthetic HSV-1 gB peptide fragments self-assemble into thioflavin-positive fibrils, form β-pleated sheets with identical appearance to Aβ by ultrastructural analysis, and accelerate *in vitro* formation of Aβ fibrils, which were neurotoxic at doses similar to Aβ. The authors describe the *in vitro* biophysical behavior of the HSV-1 gB fragment as “amyoidogenic”, and suggest that interactions between HSV-1 and Aβ may lead to gB seeding and Aβ plaque formation (Cribbs et al., 2000). Proteomic studies indicate that AD amyloid plaques and NFTs contain significant levels of HSV-1 and immune-related proteins (Carter, 2011). In addition, complement membrane attack complex is found in dystrophic neurites and NFTs in the AD brain (McGeer et al., 1989). Carter (2011) suggests that amyloid plaques are the end result of immunologic warfare between host and HSV-1. Resultant destruction of the virus is achieved at the cost of significant complement-mediated neuronal loss. The above findings are especially significant due to recent publications supporting Aβ as an antimicrobial peptide (AMP) with antiviral activity against HSV-1 as discussed below (Bourgade et al., 2015, 2016).

## Aβ Functions as An Antimicrobial Peptide (AMP)

AMPs are proteins that demonstrate potent antimicrobial effects against pathogens including viruses, bacteria and fungi (Izadpanah and Gallo, 2005). As part of the innate immune system, they can kill microbes through various mechanisms. The AMP known as eosinophil cationic protein (ECP) can self-aggregate to entrap and kill Gram-negative bacteria by agglutination (Torrent et al., 2012). Amyloid proteins and several AMPs have comparable biophysical characteristics including similar β-sheet structures and similar abilities to form fibrils, insert into cell membranes, and form channels toxic to cells (Kagan et al., 2012). Human LL-37 is a member of the cathelicidin group of AMPs, which form linear α-helix structures with hydrophobic and cationic domains. LL-37 has broad spectrum antimicrobial activity, induces angiogenesis, and is a chemoattractant of neutrophils, monocytes, and T cells (Izadpanah and Gallo, 2005). The AMP human β-defensin-1 (hBD-1) peptide localizes to areas of granulovacuolar degeneration within AD hippocampal neurons. Increased expression of hBD-1 has been demonstrated in choroid plexus brain samples from AD subjects compared to age matched controls (Williams et al., 2013).

### Aβ Demonstrates Antimicrobial Activity Against HSV-1 and Influenza A Virus

Aβ_1–42_ added to human neuronal-glial cell cultures inhibited HSV-1–induced upregulation of host cell micro-RNA-146a (miRNA-146a) levels, which are normally produced as an immune response to the virus. Aβ also decreased HSV-1 infectivity and reduced HSV-1-related pathological morphology in neuronal cells (Lukiw et al., 2010). Aβ_1–42_ and Aβ_1–40_ inhibit HSV-1 replication in fibroblasts, epithelial cells, and neuronal cells when added simultaneously or 2 h prior to HSV-1 infection. This Aβ-mediated anti-viral effect was not seen when Aβ was added to the non-enveloped human adenovirus. Experiments using a cell-free system with fluorescence detection assays indicate that Aβ peptide interacts with the HSV-1 envelope extracellularly and interferes with viral attachment and/or fusion to host cell membranes (Bourgade et al., 2015). The authors suggest that these findings, along with the shared peptide homology between Aβ and HSV-1 envelope glycoprotein B (gB) (Cribbs et al., 2000), indicate that the Aβ effect on HSV-1 replication may involve the insertion of Aβ into the viral envelope, which prevents entry of the virus into the host cell (Bourgade et al., 2015). In co-culture experiments using neuroglioma and glioblastoma cells, Aβ_1–42_ was produced by neuroglioma cells in response to infection by HSV-1. Conditioned medium containing the Aβ_1–42_ provided protection against HSV-1 replication in *de novo* neuronal cells exposed to the virus. Glioblastoma cells in co-culture were seen to internalize Aβ_1–42_ and produce cytokines IL-1β, TNF-α and IFN-α in response to combined HSV-1 and Aβ exposure (Bourgade et al., 2016).

Aβ also demonstrates AMP activity against H3N2 and H1N1 strains of influenza A virus. Aβ interfered with viral infectivity of epithelial cells and reduced viral replication, which was demonstrated by quantitative PCR. Light transmission assays and electron and confocal microscopy revealed Aβ-induced aggregation of influenza viral particles. Aβ increased monocyte phagocytosis and neutrophil uptake of the virus. There was reduction in viral protein synthesis and production of IL-6 within monocytes. Aβ_1–42_ demonstrated greater antiviral activity than Aβ_1–40_ (White et al., 2014).

### Aβ Antimicrobial Activity Against Bacteria and Yeast

Aβ has been shown to be an AMP *in vitro* against eight pathogens, including bacteria such as *Escherichia coli, Streptococcus pneumonia* and *Staphylococcus aureus*, and the fungus *Candida albicans*. Aβ demonstrates AMP activity greater than or equal to that of LL-37 against most of these pathogens. Whole brain homogenates from AD brains have significantly higher antimicrobial activity compared to samples from age-matched non-AD controls, an effect that correlates with Aβ tissue levels (Soscia et al., 2010). Aβ_x-42_ peptides of different lengths agglutinated the bacterium *Escherichia* *coli*, *Enterococcus fecalis, Listeria monocytogenes*, and *Staphylococcus aureus* and the yeast *C. albicans*. Aβ_1–42_ exhibited AMP activity against all microbes tested and killed up to 80% of pathogens within 6 h of exposure (Spitzer et al., 2016).

Aβ expressed in 5XFAD transgenic mouse, nematode *Caenorhabditis elegans* and cultured mammalian host cell monolayer AD models demonstrates protection against infection by *Salmonella typhimurium* compared to non-transgenic controls. Reduced infection by *Candida albicans* was also seen in transgenic nematode and transformed host cell models, which overexpress Aβ. Cell culture experiments implicate soluble Aβ oligomer binding to carbohydrates of the pathogen cell wall mediated by an Aβ heparin-binding motif. Aβ reduced microbial adhesion to host cells and entrapped microbes by Aβ fibril formation and agglutination. Brain infection of transgenic mice by *Salmonella typhimurium* resulted in Aβ deposition with bacteria embedded in deposits of Aβ. Transgenic Aβ-expressing mice had significantly improved clinical outcomes and survival compared to non-transgenic mice after brain infection by *S. typhimurium*. Aβ-expressing nematodes and cultured transformed Aβ-expressing mammalian cells showed improved survival after infection by *S. typhimurium* and *Candida albicans* (Kumar D. K. et al., 2016).

Bourgade et al. (2016) hypothesize that Aβ peptides are produced by neuronal cells under homeostatic conditions to perform normal physiologic functions such as synaptic plasticity and baseline antimicrobial defense. Overproduction of Aβ occurs in response to episodes of HSV-1 reactivation in the brain, as well as other CNS infections and pathological insults. This leads to fibrillization of Aβ and formation of amyloid plaques. CNS infection and Aβ deposition activate microglia, resulting in cytokine overproduction and a vicious cycle of neuronal damage and neurodegeneration (Bourgade et al., 2016). The concept that Aβ functions as an AMP is further substantiated by the presence of amyloid plaques in other neurodegenerative diseases associated with pathogens.

## Cerebral Amyloid Plaques in Other Brain Infections and Infection-Related Dementias

**Figure 7:**
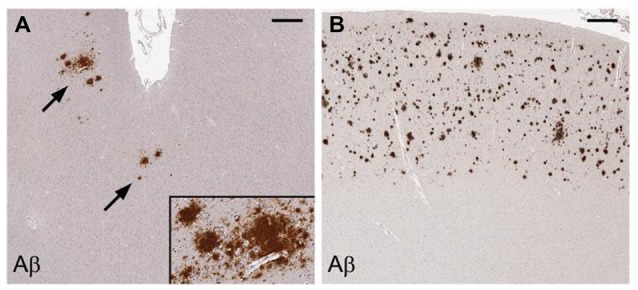
Cerebral amyloid plaques containing Aβ in middle frontal cortex samples from HIV-infected patients. Immunohistochemical staining with anti-Aβ antibody demonstrates scattered focal plaques (**A**, arrows) and widespread plaques **(B)** in the cortex. Scale bars = 500 μm. Figure from Soontornniyomkij et al. (2012). Color version of figure from HHS Public Access PMCID: PMC3576852. Reprinted with permission from Wolters Kluwer Health, Inc.

HIV patients dying between ages 30–69 have a significantly increased prevalence of diffuse largely non-neuritic amyloid plaques in brain samples from the frontal and temporal cortices than age matched, non-HIV-infected controls (Esiri et al., 1998). Another postmortem study has shown significantly increased extracellular and intraneuronal cerebral Aβ plaques in AIDS patients previously treated with highly active anti-retroviral therapy (HART) compared to age and sex-matched controls who did not have access to antiviral treatment. Plaques increased with age in both groups. Nearly 50% of the AIDS brains in the study were found to have Aβ deposition in the frontal cortex. Mechanisms proposed by the authors to explain these findings include the persistence of HIV in brain despite treatment with HART, possible HART-related inhibition of insulin degrading enzyme, and HART-related inhibition of APP axonal transport (Green et al., 2005). Diffuse cerebral Aβ plaques (Figure 7) are associated with HAND in subjects who possess the *APOE*-ε4 allele (adjusted OR = 30.0), but not in *APOE*-ε4-negative subjects (Soontornniyomkij et al., 2012).

**Figure 8:**
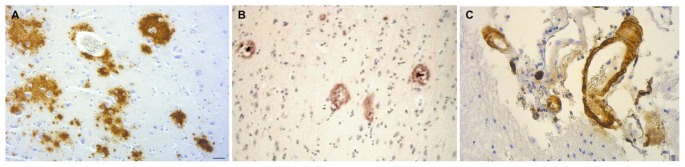
Amyloid deposits containing Aβ in brain samples from neurosyphilis patients. **(A)** Cortical amyloid deposits from patients diagnosed with dementia due to neurosyphilis showing positive immunoreaction with anti-Aβ 8–17 (6F/3D, DakoCytomation) antibody. **(B)** Aβ deposition resembling immature and mature plaques. **(C)** Aβ deposits seen in the arterial wall of leptomeningeal vessels in the same patient as **(A)**. Immunohistochemical analysis of Aβ was performed using the avidin-biotine-peroxidase technique. Bar = 50 μm. Panels **(A)** and **(C)** were reproduced from Figure 2 of Miklossy et al. (2006b). Figure from Miklossy (2015). Reprinted under the terms of the Creative Commons Attribution License (CC BY).

Cerebral amyloid plaques are seen in dementia patients with chronic bacterial infections. *C. pneumoniae*-infected cells identified in four AD postmortem brains co-localized with senile amyloid plaques and NFTs identified by immunostaining with monoclonal antibodies (Gérard et al., 2006). Evaluation of postmortem brain samples from syphilis patients with the confirmed diagnosis of general paresis caused by *T. pallidum* revealed Aβ deposition with similar appearance to immature and mature amyloid plaques found in AD (Figure 8; Miklossy et al., 2006b; Miklossy, 2015). NFTs have also been found in brains of syphilitic dementia patients (Miklossy et al., 2006b; Miklossy, 2011b). *Borrelia* antigens and genes co-localized with senile plaques and with NFTs in AD brains from which *B. burgdorferi* was cultured. In addition, *Borrelia* antigens were found to specifically immunolocalize with Aβ (Miklossy et al., 2004). Bacterial peptidoglycan has been found to co-localize with Aβ, senile plaques, and NFTs using immunohistochemistry techniques in AD postmortem brain specimens (Miklossy et al., 1996, 2004; Miklossy, 1998). Studies indicate that spirochetes induce formation of amyloid plaques and AD-like pathology. Infection of mammalian neurons, astrocytes, microglial cells, and brain organotypic cell aggregates *in vitro* by the spirochete *Borrelia burgdorferi* *sensu strictu* results in the formation of amyloid plaques with β-pleated sheet structure, tangle-like formations, and AD-like cellular changes. Increases in levels of AβPP and hyperphosphorylated tau proteins were detected by western blot (Miklossy et al., 2006a). The synthetic peptide BH (9–10), which corresponds to the β-hairpin segment of the *B. burgdorferi* OspA protein, forms amyloid-like fibrils *in vitro* (Ohnishi et al., 2000).

Prion protein (PrP) amyloid plaques are found in postmortem brain samples from subjects diagnosed with prion-related transmissible spongiform encephalopathies (TSEs), including Creutzfeldt-Jakob disease (CJD), hereditary Gerstmann-Straussler-Scheinker syndrome, kuru, and the animal prion disease scrapie (Liberski, 1994). Aβ-containing plaques have been identified in CJD (Barcikowska et al., 1995; Debatin et al., 2008), with mixed CJD/AD found in 2%–15% of studies involving brain bank cases. This is particularly relevant because the wall-less bacterium spiroplasma has been implicated in the pathogenesis of CJD (Bastian, 2017). In addition, prion amyloid protein has been separated from infectivity suggesting PrP aggregation may be an innate immune response to infection (Miyazawa et al., 2012).

## Epidemiologic Studies Associating HSV-1 Infection with AD and Cognitive Impairment

Studies of infectious burden (IB) use a composite measure of serum antibody levels to assess prior exposure to several pathogens. HSV-1 infection as part of IB contributes to the associated increased risk for development of MCI and AD in many of these studies. Serum IgG antibody titers to *Herpesviridae* HSV-1, HSV-2, CMV, as well as *Chlamydia pneumoniae* and *Mycoplasma pneumoniae* bacteria were measured in 383 elderly patients with cardiovascular disease. Subjects having three positive viral titers were found to have a 2.3 times higher risk for cognitive impairment after 12 months. Bacterial IB did not associate with cognitive impairment (Strandberg et al., 2003). Katan et al. (2013) studied 1625 subjects with seropositive evidence for exposure to HSV-1, HSV-2, CMV, *Chlamydia pneumoniae* and *Helicobacter pylori*, and found that IB was associated with cognitive impairment. The findings appeared to be determined primarily by viral IB (Katan et al., 2013; Strandberg and Aiello, 2013). Gale et al. (2016) studied 5662 young to middle-aged adults, and found that subjects with IgG seropositivity to HSV-1, CMV, and hepatitis A had the most significant cognitive decline compared to subjects seropositive to HSV-2, hepatitis B, hepatitis C, toxoplasmosis and toxocariasis. Bu et al. (2015) demonstrated that higher viral IB (HSV-1 and CMV), bacterial IB (*B. burgdorferi, C. pneumoniae* and *H. pylori*), and total IB independently associated with AD after adjusting for *APOE* genotype, age, gender, education and other comorbidities. Subjects with higher IB had higher levels of serum Aβ and higher levels of serum proinflammatory cytokines including IFN-γ, TNF-α, IL-1β and IL-6. AD patients with higher IB also demonstrated higher serum Aβ and cytokine levels.

HSV-1 reactivation as measured by the presence of baseline anti-HSV-1 immunoglobulin M (IgM) antibodies is associated with increased risk of developing AD. In one study, 512 elderly subjects initially without dementia were followed for 14 years. Baseline positive HSV-1 IgM seropositivity increased the risk of developing AD by a factor of 2.55 (Letenneur et al., 2008). Similar results were obtained in study by Lövheim et al. (2015a) who followed 3422 subjects for average follow up time of 11.3 years. Baseline HSV-1 IgM seropositivity increased AD risk by a factor of 1.95. Kobayashi et al. (2013) used anti-HSV-1 IgG antibody avidity index, a measure of the strength to which IgG attaches to viral antigen, as an indicator of HSV-1 reactivation in a study involving patients with amnestic MCI (aMCI) and AD. Patients with aMCI had higher HSV-1 IgG antibody avidity index levels than AD patients and healthy controls. The results indicate that HSV-1 reactivation occurs more often in the aMCI group and suggest that HSV-1 reactivation contributes to development of aMCI (Kobayashi et al., 2013).

Lifelong infection with HSV-1, as measured by the presence of anti-HSV IgG antibodies, is associated with increased risk for developing AD. A longitudinal study involving 360 patients with average age at baseline 61.2 years followed for 6.6 years or longer demonstrated that baseline positive HSV-1 IgG antibody levels increased the risk for developing AD by a factor of 2.25 (Lövheim et al., 2015b). Mancuso et al. (2014) found that a strong humoral response in AD patients, as indicated by higher HSV-1 IgG titers, was associated with preservation of orbitofrontal and bilateral temporal cortical gray matter volumes measured on brain MRI. Agostini et al. (2016) corroborated the protective nature of a higher HSV-1 humoral response by finding significantly higher baseline HSV-1 IgG antibody titers and antibody avidity in aMCI-non-converters compared to aMCI-converters. Higher HSV-1 antibody levels were also associated with better-preserved left hippocampus and amygdala cortical volumes as measured by brain MRI.

HSV-1 IgG seropositivity is also associated with increased risk of cognitive impairment in younger healthy subjects ages 17–21 (Fruchter et al., 2015) and across all age groups (Tarter et al., 2014) when compared to HSV-1 IgG seronegative controls. Various measures of cognition are impaired in several studies involving middle-aged HSV-1 IgG positive schizophrenic patients compared to schizophrenic HSV-1 IgG negative controls (Dickerson et al., 2003, 2012; Shirts et al., 2008; Schretlen et al., 2010; Yolken et al., 2011; Prasad et al., 2012). HSV-1 infection does not associate with increased risk for schizophrenia; however, exposure to the virus does associate with impaired cognition in this cohort of neuropsychiatric patients (Schretlen et al., 2010; Prasad et al., 2012). HSV-1 seropositivity also associates with decreased gray matter volume on MRI in the prefrontal cortex, anterior cingulate cortex, and areas of cerebellum in these patients (Prasad et al., 2007; Schretlen et al., 2010).

A recent meta-analysis of research publications involving *Herpesviridae* and AD evaluated eighteen HSV-1 related studies. Combined results from studies measuring HSV-1 antibody serology or HSV-1 DNA from brain showed that infection with HSV-1 alone (OR = 1.38) and in combination with the *APOE-ε4* allele (OR = 2.25) significantly increased the risk of AD (Steel and Eslick, 2015).

## Evidence for HSV-1 Reactivation in the Brain

Methodology is lacking to directly detect the hypothesized periodic limited subclinical reactivation of latent HSV-1 in the AD brain (Itzhaki, 2014, 2017). However, several studies indirectly support the hypothesis. HSV-1 reactivation as measured by the presence of baseline anti-HSV-1 IgM antibodies is associated with increased risk of developing AD (Letenneur et al., 2008; Lövheim et al., 2015a). Saldanha et al. (1986) found HSV-1 DNA sequences at levels detectable by *in situ* hybridization—evidence for reactivation—in postmortem brain samples from immunosuppressed leukemic patients with serological evidence of past HSV-1 infection. HSV-1 DNA was not found in brains from non-immunosuppressed and HSV-1 seronegative patients. HSV-1 DNA and antigens were identified in the cytoplasm of cortical neurons from three patients with familial AD indicating viral replication likely due to reactivation of the virus (Mori et al., 2004). Klapper et al. (1984) suggests an underdiagnosed subacute form of HSE. Mild forms of HSE have been described with less severe symptoms and better prognosis (Klapper et al., 1984; Marton et al., 1995).

HSV-1 reactivation from latency in brains of immune deficient mice has been demonstrated *in vivo* (Ramakrishna et al., 2015). HSV-1 latently infected neuronal cells from mouse brains were shown to reactivate by modified *ex-vivo* culture methods (Chen et al., 2006; Yao et al., 2014). Reactivation of the virus was also demonstrated in latently infected brain tissue from tree shrews using similar explant culture techniques (Li et al., 2016). Neuronal ICP4 viral antigen expression—indicating HSV-1 reactivation from latency—was associated with molecular indicators of neuroinflammation and early neurodegeneration in the cerebral cortices of asymptomatic HSV-1 infected mice (Martin et al., 2014).

## HSV-1 Reactivation in the Peripheral Nervous System

Reactivation of HSV-1 does occur in the peripheral nervous system, with studies suggesting that not all virions within groups of neurons are quiescent during latency. Asymptomatic HSV-1 reactivation and shedding in human tears and saliva occurred at a high rate (98%) in HSV seropositive adults without signs of ocular herpetic disease during a 30-day study (Kaufman et al., 2005). Elevated levels of cytokines and chemokines are found within latently infected human and mouse TG (Cantin et al., 1995; Halford et al., 1996; Held and Derfuss, 2011). Analysis by *in situ* hybridization within HSV-1-infected mouse TG during latency revealed viral DNA, viral transcripts, and viral proteins within rare neurons without detection of infectious virions. This process occurred in one neuron per 10 latently infected mouse trigeminal ganglia, which is equivalent to about one neuron expressing high-level productive cycle viral genes in each ganglion every 10 days. The authors suggest that the resulting continuous antigenic stimulus promotes an immune response characterized by focal white cell infiltrates commonly seen surrounding latently infected TG (Feldman et al., 2002). Margolis et al. (2007) found viral protein expression, positive HSV-1 antigen staining, and infectious virus in 6% of “latently” infected murine trigeminal ganglia. Immunohistochemical staining revealed associated neuronal loss and viral spreading to adjacent cells. Thus, in a mouse TG model, HSV-1 continuously reactivates as a “localized incomplete or low level lytic infection”. Active infection in a small percentage of neurons at any given time results in a persistent immune response (Conrady et al., 2010).

## Rationale for An Antiviral AD Clinical Trial

The antiviral medication acyclovir is a nucleoside analog, which is activated through phosphorylation by viral thymidine kinase and cellular kinases. The resultant acyclo-guanosine triphosphate interferes with HSV-1 DNA replication by incorporating into viral DNA and inducing premature chain termination (Elion, 1982). Treatment with acyclovir significantly reduced T cell expression of IFN-γ mRNA and TNF-α mRNA in TG from mice latently infected with HSV-1 compared to untreated latently infected controls. The authors suggest that this is likely due to decreased viral replication and antigen production (Halford et al., 1997). Sawtell et al. (1999) used a murine hyperthermic stress (HS) model of *in vivo* HSV-1 reactivation to show that acyclovir treatment blocked the production of infectious virus within latently infected mouse ganglia by >90%. Thus, acyclovir inhibits viral replication during reactivation with the potential for decrease in viral spreading.

**Figure 9:**
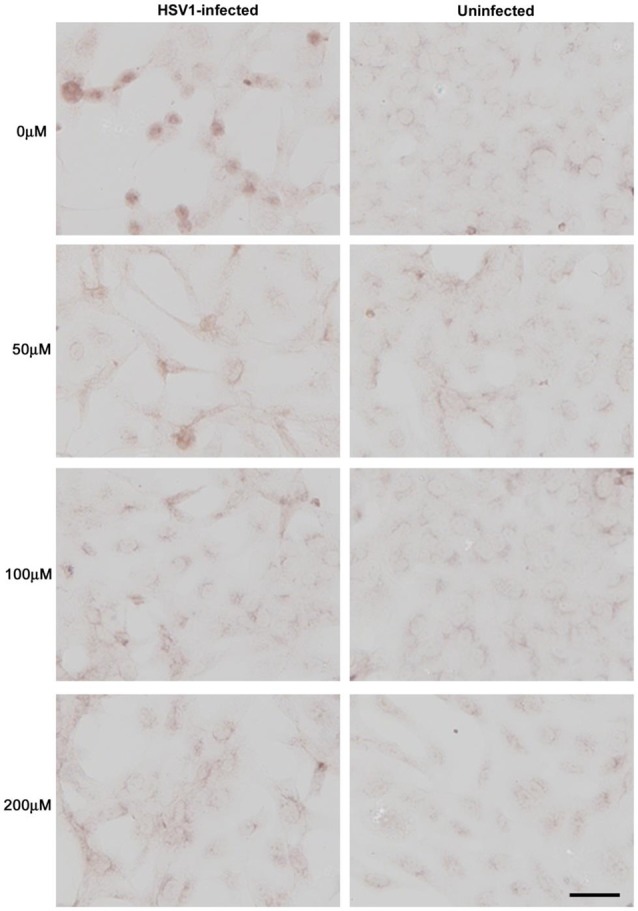
Acyclovir reduces Aβ accumulation in HSV-1-infected Vero cells. Vero cells were infected with HSV-1 SC16 at a MOI of 1 for 16 h. Cells were treated with 0 μM, 50 μM, 100 μM or 200 μM acyclovir. After fixation, immunocytochemistry was used to test the slides for Aβ accumulation. Acyclovir significantly reduced HSV-1-induced Aβ accumulation. Scale bar = 50 μm. Figure from Wozniak et al. (2011). Reprinted under the terms of the Creative Commons Attribution License (http://creativecommons.org/licenses/by/2.0).

**Figure 10:**
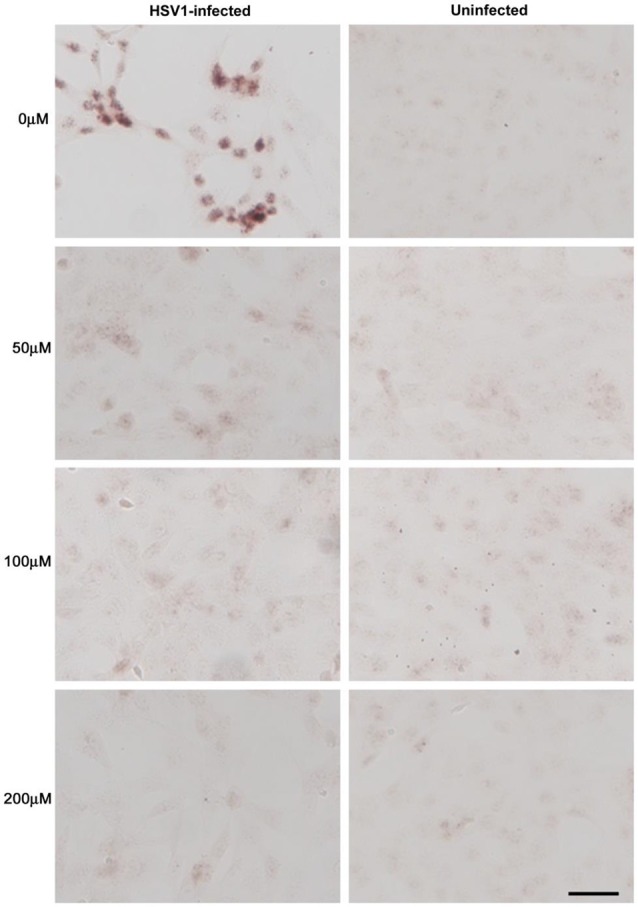
Acyclovir reduces abnormal tau phosphorylation in HSV-1-infected Vero cells. Vero cells were infected with HSV-1 SC16 at a MOI of 1 for 16 h. Cells were treated with 0 μM, 50 μM, 100 μM or 200 μM acyclovir. After fixation, immunocytochemistry was used to test the slides for abnormal tau phosphorylation. Acyclovir significantly reduced AT100 staining, indicating inhibition of HSV-1-induced abnormal tau phosphorylation. Scale bar = 50 μm. Figure from Wozniak et al. (2011). Reprinted under the terms of the Creative Commons Attribution License (http://creativecommons.org/licenses/by/2.0).

**Figure 11:**
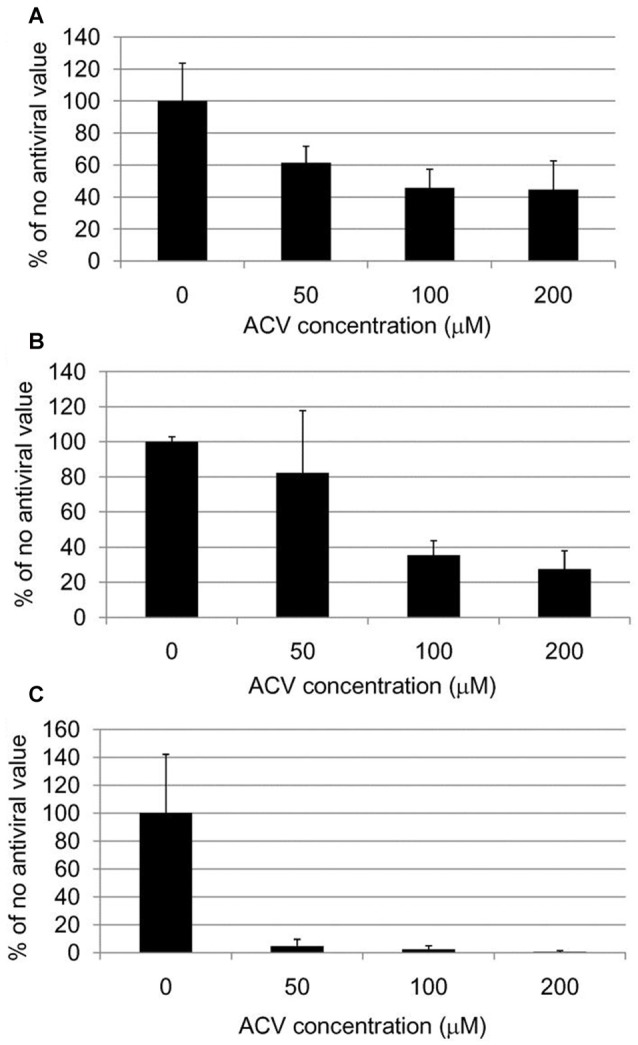
Quantification of HSV-1 proteins, β-amyloid and phosphorylated tau proteins in HSV-1-infected Vero cells following treatment with acyclovir. Vero cells were infected with HSV-1 SC16 at a MOI of 1 for 16 h. Cells were treated with 0 μM, 50 μM, 100 μM or 200 μM acyclovir. After fixation, immunocytochemistry was used to test the slides for HSV-1 proteins, Aβ accumulation, and abnormal tau phosphorylation. Values are presented as the percentage of staining detected when no acyclovir is used. Statistically significant decreases in staining for HSV-1 proteins **(A)** and abnormal tau phosphorylation **(C)** are seen with all acyclovir concentrations tested compared to cells infected but not treated with acyclovir (*p* < 0.0001 in both cases). Statistically significant decreases in Aβ staining **(B)** are seen with acyclovir concentrations of 100 μM and 200 μM (*p* < 0.0001). Figure from Wozniak et al. (2011). Reprinted under the terms of the Creative Commons Attribution License (http://creativecommons.org/licenses/by/2.0).

Treatment of HSV-1-infected Vero cells with acyclovir resulted in reductions in HSV-1-induced Aβ accumulation by 70% and inhibition of abnormal tau phosphorylation by nearly 100%, with results statistically significant compared to infected untreated cells. Acyclovir inhibited viral replication as shown by significant reductions in viral protein levels (Figures 9–11; Wozniak et al., 2011). Acyclovir reduced Aβ production by decreasing viral spreading, while phosphorylated tau reduction was attributable to antiviral inhibition of HSV-1 DNA replication. The antiviral medications penciclovir and foscarnet also reduced Aβ and phosphorylated tau accumulation in infected cell cultures. Antiviral medications were seen to reduce HSV-1-induced increases in β-secretase, nicastrin (a component of the γ-secretase complex), PKA, and GSK3β, which are enzymes or enzyme components involved in the production of Aβ and/or phosphorylation of tau proteins (Wozniak et al., 2011).

Acyclovir and valacyclovir, the better-absorbed prodrug of acyclovir, are commonly prescribed for the treatment of HSV infections (Smith et al., 2010). After oral administration, valacyclovir is rapidly hydrolyzed to acyclovir by first pass metabolism in the intestine and liver. Subsequently, acyclovir crosses the BBB attaining CSF levels required to treat HSV infections in the CNS (Lycke et al., 2003; Smith et al., 2010). Herpes simplex encephalitis has been successfully treated with valacyclovir (Pouplin et al., 2011). Chronic treatment with acyclovir and valacyclovir reduces the number of HSV outbreaks in patients with recurrent genital herpes (Goldberg et al., 1993; Tyring et al., 2002). Studies have shown that prophylactic acyclovir administration can decrease asymptomatic viral shedding in humans (Sawtell et al., 1999). Valacyclovir is also used for HSV suppression in immunocompromised patients. Sensitivity studies indicate a low rate of HSV resistance to acyclovir (<0.5%) when used in immunocompetent patients. These medications have demonstrated safety during long-term use with a mild side effect profile (Tyring et al., 2002). Reversible neuropsychiatric symptoms have been reported infrequently during treatment and are usually associated with pre-existing renal insufficiency (Smith et al., 2010). Patients with abnormal renal function require dose adjustments with these medications (Martinez-Diaz and Hsia, 2011). Twenty-nine multiple sclerosis patients treated with valacyclovir at a dose of 3 grams per day for 2 years had no discontinuation of the medication due to side effects in a clinical trial by Friedman et al. (2005).

Interestingly, a randomized controlled clinical trial involving 24 HSV-1 IgG seropositive schizophrenia patients treated with valacyclovir for 18 weeks showed significant improvement in verbal memory, working memory, and visual object learning when compared to a non-treated HSV-1 IgG seropositive schizophrenia control group. Both groups were taking anti-psychotic medication. While psychotic symptoms did not improve, this study did demonstrate improved cognition in HSV-1-infected neuropsychiatric patients treated with antiviral medication (Prasad et al., 2013).

## Conclusion

Animal and *in vitro* studies reveal numerous mechanisms whereby HSV-1 is able to induce cellular processes involved in AD pathogenesis, including neuronal production of Aβ, hyperphosphorylation of tau protein, dysregulation of calcium homeostasis, and impaired autophagy. In addition, the virus causes neuroinflammation, oxidative stress, mitochondrial damage, synaptic dysfunction and neuronal apoptosis. Pathogenic effects by HSV-1 replicate key aspects of AD pathophysiology.

HSV-1 interacts with AD-related genes and proteins to induce AD pathogenesis. Carriage of *APOE*-ε4 increases HSV-1 viral load in the brain (Burgos et al., 2006) and increases the innate immune response (Gale et al., 2014). Additional AD susceptibility genes, including *CR1*, *CLU*, *PICALM* and *NC-2*, are involved in the HSV-1 lifecycle. Polymorphisms in these genes may affect susceptibility to brain infection by herpes viruses, triggering AD-related pathology (Porcellini et al., 2010; Licastro et al., 2011). Infection by HSV-1 alters neuronal gene expression for neprilysin and modulates enzyme activity for neprilysin and GSK3β—key enzymes involved in Aβ deposition and hyperphosphorylation of tau protein (Civitelli et al., 2015). HSV-1 infection of neuronal cells also alters expression of genes affecting cognition-related pathways, including CREB, glutamate receptor signaling, and voltage-gated ion channels (D’Aiuto et al., 2015). Host immune response to HSV-1 by *CH25H* may promote Aβ deposition (Itzhaki et al., 2016) as well as AD-related atherosclerosis and vascular occlusion (Lathe et al., 2014).

Evidence supporting Aβ as an AMP against viral, bacterial and fungal pathogens (Lukiw et al., 2010; Soscia et al., 2010; Bourgade et al., 2015) may change the paradigm regarding AD pathophysiology. In the case of HSV-1, research data suggests that Aβ interferes with viral attachment or fusion to neuronal cells, which inhibits viral replication (Bourgade et al., 2015). Increased neuronal production of Aβ in response to HSV-1 and other infections and insults in the brain could tip the balance from lower, homeostatic Aβ levels towards Aβ accumulation and plaque formation in individuals genetically susceptible to AD (Bourgade et al., 2016).

Human and animal studies support the hypothesized reactivation of latent HSV-1 in the AD brain. Localized subacute reactivation of HSV-1 in the brain is consistent with the slowly progressive course in sporadic AD. The resultant damage from low level viral spread, antigenic stimulation, and innate immune response provides the necessary stimulus to initiate and perpetuate uncontrolled neuroinflammation and neurodegeneration, as proposed by Gao and Hong (2008). Peripheral HSV-1 reactivation and immune response may also contribute to adaptive immune system involvement in AD pathogenesis as proposed by Lynch (2014).

An antiviral clinical trial using valacyclovir in HSV-1 IgG seropositive patients with MCI or AD, especially *APOE*-ε4 carriers, has been proposed as part of a comprehensive antimicrobial AD research strategy (Itzhaki et al., 2016). The medication acts on HSV-1-infected cells only (Wozniak et al., 2011), exhibits a low side effect profile, and demonstrates safety with chronic use (Tyring et al., 2002). By halting the direct and indirect toxic effects of HSV-1 on neuronal cells, antiviral medication may play a role in the prevention and treatment of AD. Furthermore, a mixed glycoprotein HSV-1 vaccine has been shown to be effective in reducing HSV-1 in mouse brain after peripheral infection (Lin et al., 2001). Although not yet developed, a human HSV-1 vaccine may prove beneficial in the prevention of AD by reducing primary infection and reactivation of the virus.

## Author Contributions

SAH and EAH initiated the work and contributed to the conception, design, analysis and reproduction of the data. They wrote the manuscript, prepared the illustrations, and take responsibility for the accuracy and integrity of the presented work.

## Conflict of Interest Statement

The authors declare that the research was conducted in the absence of any commercial or financial relationships that could be construed as a potential conflict of interest.

## References

[B1] Agostini S. Mancuso R. Baglio F. Cabinio M. Hernis A. Costa A. S.. (2016). High avidity HSV-1 antibodies correlate with absence of amnestic Mild Cognitive Impairment conversion to Alzheimer’s disease. Brain Behav. Immun. 58, 254–260. 10.1016/j.bbi.2016.07.153 27470229

[B2] Alexander D. E. Leib D. A. (2008). Xenophagy in herpes simplex virus replication and pathogenesis. Autophagy 4, 101–103. 10.4161/auto.5222 18000391 PMC3775468

[B3] Alonso R. Pisa D. Marina A. I. Morato E. Rábano A. Carrasco L. (2014a). Fungal infection in patients with Alzheimer’s disease. J. Alzheimers Dis. 41, 301–311. 10.3233/JAD-132681 24614898

[B4] Alonso R. Pisa D. Rábano A. Carrasco L. (2014b). Alzheimer’s disease and disseminated mycoses. Eur. J. Clin. Microbiol. Infect. Dis. 33, 1125–1132. 10.1007/s10096-013-2045-z 24452965

[B5] Alonso R. Pisa D. Rábano A. Rodal I. Carrasco L. (2015). Cerebrospinal fluid from Alzheimer’s disease patients contains fungal proteins and DNA. J. Alzheimers Dis. 47, 873–876. 10.3233/JAD-150382 26401766

[B254] Alonso Vilatela M. E. López-López M. Yescas-Gómez P. (2012). Genetics of Alzheimer’s disease. Arch. Med. Res. 43, 622–631. 10.1016/j.arcmed.2012.10.017 23142261

[B6] Alvarez G. Aldudo J. Alonso M. Santana S. Valdivieso F. (2012). Herpes simplex virus type 1 induces nuclear accumulation of hyperphosphorylated tau in neuronal cells. J. Neurosci. Res. 90, 1020–1029. 10.1002/jnr.23003 22252837

[B7] Anthony I. C. Ramage S. N. Carnie F. W. Simmonds P. Bell J. E. (2006). Accelerated tau deposition in the brains of individuals infected with human immunodeficiency virus-1 before and after the advent of highly active anti-retroviral therapy. Acta Neuropathol. 111, 529–538. 10.1007/s00401-006-0037-0 16718349

[B8] Armien A. G. Hu S. Little M. R. Robinson N. Lokensgard J. R. Low W. C.. (2010). Chronic cortical and subcortical pathology with associated neurological deficits ensuing experimental herpes encephalitis. Brain Pathol. 20, 738–750. 10.1111/j.1750-3639.2009.00354.x 20002440 PMC2895975

[B9] Athmanathan S. Vydehi B. V. Sundaram C. Vemuganti G. K. Murthy J. M. (2001). Neuronal apoptosis in herpes simplex virus—1 encephalitis (HSE). Indian J. Med. Microbiol. 19, 127–131. Available online at: http://www.ijmm.org/text.asp?2001/19/3/127/8145 17664814

[B10] Aurelius E. Andersson B. Forsgren M. Sköldenberg B. Strannegård O. (1994). Cytokines and other markers of intrathecal immune response in patients with herpes simplex encephalitis. J. Infect. Dis. 170, 678–681. 10.1093/infdis/170.3.678 8077727

[B11] Bales K. R. Dodart J. C. DeMattos R. B. Holtzman D. M. Paul S. M. (2002). Apolipoprotein E, amyloid, and Alzheimer disease. Mol. Interv. 2, 363–375, 339. 10.1124/mi.2.6.363 14993413

[B12] Balin B. J. Gérard H. C. Arking E. J. Appelt D. M. Branigan P. J. Abrams J. T.. (1998). Identification and localization of Chlamydia pneumoniae in the Alzheimer’s brain. Med. Microbiol. Immunol. 187, 23–42. 10.1007/s004300050071 9749980

[B13] Balin B. J. Little C. S. Hammond C. J. Appelt D. M. Whittum-Hudson J. A. Gérard H. C.. (2008). Chlamydophila pneumoniae and the etiology of late-onset Alzheimer’s disease. J. Alzheimers Dis. 13, 371–380. 10.3233/jad-2008-13403 18487846

[B14] Ball M. J. (1982). “Limbic predilection in Alzheimer dementia: is reactivated herpes virus involved?”. Can. J. Neurol. Sci. 9, 303–306. 10.1017/s0317167100044115 7116237

[B16] Banks W. A. (2005). Blood-brain barrier transport of cytokines: a mechanism for neuropathology. Curr. Pharm. Des. 11, 973–984. 10.2174/1381612053381684 15777248

[B15] Banks W. A. Farr S. A. La Scola M. E. Morley J. E. (2001). Intravenous human interleukin-1α impairs memory processing in mice: dependence on blood-brain barrier transport into posterior division of the septum. J. Pharmacol. Exp. Ther. 299, 536–541. 11602664

[B17] Barcikowska M. Kwiecinski H. Liberski P. P. Kowalski J. Brown P. Gajdusek D. C. (1995). Creutzfeldt-Jakob disease with Alzheimer-type A β-reactive amyloid plaques. Histopathology 26, 445–450. 10.1111/j.1365-2559.1995.tb00252.x 7657313

[B18] Baringer J. R. Pisani P. (1994). Herpes simplex virus genomes in human nervous system tissue analyzed by polymerase chain reaction. Ann. Neurol. 36, 823–829. 10.1002/ana.410360605 7998767

[B19] Bastian F. O. (2017). Combined creutzfeldt-jakob/ Alzheimer’s disease cases are important in search for microbes in Alzheimer’s disease. J. Alzheimers Dis. 56, 867–873. 10.3233/JAD-160999 28059790

[B20] Bertrand P. Guillaume D. Hellauer L. Dea D. Lindsay J. Kogan S. (1993). Distribution of herpes simplex virus type 1 DNA in selected areas of normal and Alzheimer’s disease brains: a PCR study. Neurodegeneration 2, 201–208.

[B21] Bezprozvanny I. Mattson M. P. (2008). Neuronal calcium mishandling and the pathogenesis of Alzheimer’s disease. Trends Neurosci. 31, 454–463. 10.1016/j.tins.2008.06.005 18675468 PMC2566585

[B22] Blanc M. Hsieh W. Y. Robertson K. A. Kropp K. A. Forster T. Shui G.. (2013). The transcription factor STAT-1 couples macrophage synthesis of 25-hydroxycholesterol to the interferon antiviral response. Immunity 38, 106–118. 10.1016/j.immuni.2012.11.004 23273843 PMC3556782

[B23] Block M. L. Zecca L. Hong J. S. (2007). Microglia-mediated neurotoxicity: uncovering the molecular mechanisms. Nat. Rev. Neurosci. 8, 57–69. 10.1038/nrn2038 17180163

[B24] Boelen E. Steinbusch H. W. Pronk I. Grauls G. Rennert P. Bailly V.. (2007). Inflammatory responses following Chlamydia pneumoniae infection of glial cells. Eur. J. Neurosci. 25, 753–760. 10.1111/j.1460-9568.2007.05339.x 17313571

[B25] Boland B. Kumar A. Lee S. Platt F. M. Wegiel J. Yu W. H.. (2008). Autophagy induction and autophagosome clearance in neurons: relationship to autophagic pathology in Alzheimer’s disease. J. Neurosci. 28, 6926–6937. 10.1523/JNEUROSCI.0800-08.2008 18596167 PMC2676733

[B26] Bonda D. J. Wang X. Perry G. Nunomura A. Tabaton M. Zhu X.. (2010). Oxidative stress in Alzheimer disease: a possibility for prevention. Neuropharmacology 59, 290–294. 10.1016/j.neuropharm.2010.04.005 20394761

[B27] Bourgade K. Garneau H. Giroux G. Le Page A. Y. Bocti C. Dupuis G.. (2015). β-Amyloid peptides display protective activity against the human Alzheimer’s disease-associated herpes simplex virus-1. Biogerontology 16, 85–98. 10.1007/s10522-014-9538-8 25376108

[B28] Bourgade K. Le Page A. Bocti C. Witkowski J. M. Dupuis G. Frost E. H.. (2016). Protective effect of amyloid-β peptides against herpes simplex virus-1 infection in a neuronal cell culture model. J. Alzheimers Dis. 50, 1227–1241. 10.3233/JAD-150652 26836158

[B29] Braak H. Braak E. Bohl J. (1993). Staging of Alzheimer-related cortical destruction. Eur. Neurol. 33, 403–408. 10.1159/000116984 8307060

[B30] Brown L. A. Scarola J. Smith A. J. Sanberg P. R. Tan J. Giunta B. (2014). The role of tau protein in HIV-associated neurocognitive disorders. Mol. Neurodegener. 9:40. 10.1093/med/9780199937837.003.0147 25304757 PMC4210623

[B31] Bu X. L. Yao X. Q. Jiao S. S. Zeng F. Liu Y. H. Xiang Y.. (2015). A study on the association between infectious burden and Alzheimer’s disease. Eur. J. Neurol. 22, 1519–1525. 10.1111/ene.12477 24910016

[B32] Burgos J. Ramirez C. Sastre I. Valdivieso F. (2006). Effect of Apolipoprotein E on the cerebral load of latent herpes simplex virus type 1 DNA. J. Virol. 80, 5383–5387. 10.1128/jvi.00006-06 16699018 PMC1472141

[B33] Burt T. D. Agan B. K. Marconi V. C. He W. Kulkarni H. Mold J. E.. (2008). Apolipoprotein (apo) E4 enhances HIV-1 cell entry *in vitro* and the *APOE* ɛ4/ɛ4 genotype accelerates HIV disease progression. Proc. Natl. Acad. Sci. U S A 105, 8718–8723. 10.1073/pnas.0803526105 18562290 PMC2438419

[B34] Cai Z. Hussain M. D. Yan L. J. (2014). Microglia, neuroinflammation, and β-amyloid protein in Alzheimer’s disease. Int. J. Neurosci. 124, 307–321. 10.3109/00207454.2013.833510 23930978

[B35] Cantin E. M. Hinton D. R. Chen J. Openshaw H. (1995). γ interferon expression during acute and latent nervous system infection by herpes simplex virus type 1. J. Virol. 69, 4898–4905. 7609058 10.1128/jvi.69.8.4898-4905.1995 PMC189304

[B36] Carbone I. Lazzarotto T. Ianni M. Porcellini E. Forti P. Masliah E.. (2014). Herpes virus in Alzheimer’s disease: relation to progression of the disease. Neurobiol. Aging 35, 122–129. 10.1016/j.neurobiolaging.2013.06.024 23916950

[B37] Cárdenas-Aguayo M. D. C. Silva-Lucero M. D. C. Cortes-Ortiz M. Jiménez-Ramos B. Gómez-Virgilio L. Ramírez-Rodríguez G. (2014). “Physiological role of amyloid β in neural cells: the cellular trophic activity,” in Neurochemistry, ed. Heinbockel T. (London: InTech), 257–281.

[B38] Carter C. J. (2010). APP, APOE, complement receptor 1, clusterin and PICALM and their involvement in the herpes simplex life cycle. Neurosci. Lett. 483, 96–100. 10.1016/j.neulet.2010.07.066 20674675

[B39] Carter C. J. (2011). Alzheimer’s disease plaques and tangles: cemeteries of a pyrrhic victory of the immune defence network against herpes simplex infection at the expense of complement and inflammation-mediated neuronal destruction. Neurochem. Int. 58, 301–320. 10.1016/j.neuint.2010.12.003 21167244

[B40] Castellano J. M. Kim J. Stewart F. R. Jiang H. DeMattos R. B. Patterson B. W.. (2011). Human apoE isoforms differentially regulate brain amyloid-β peptide clearance. Sci. Transl. Med. 3:89ra57. 10.1126/scitranslmed.3002156 21715678 PMC3192364

[B41] Chami L. Checler F. (2012). BACE1 is at the crossroad of a toxic vicious cycle involving cellular stress and β-amyloid production in Alzheimer’s disease. Mol. Neurodegener. 7:52. 10.1186/1750-1326-7-52 23039869 PMC3507664

[B42] Chen S. H. Yao H. W. Huang W. Y. Hsu K. S. Lei H. Y. Shiau A. L.. (2006). Efficient reactivation of latent herpes simplex virus from mouse central nervous system tissues. J. Virol. 80, 12387–12392. 10.1128/jvi.01232-06 17005636 PMC1676271

[B43] Cheng S. B. Ferland P. Webster P. Bearer E. L. (2011). Herpes simplex virus dances with amyloid precursor protein while exiting the cell. PLoS One 6:e17966. 10.1371/journal.pone.0017966 21483850 PMC3069030

[B44] Cheon M. S. Bajo M. Gulesserian T. Cairns N. Lubec G. (2001). Evidence for the relation of herpes simplex virus type1 to Down syndrome and Alzheimer’s disease. Electrophoresis 22, 445–448. 10.1002/1522-2683(200102)22:3<445::aid-elps445>3.0.co;2-8 11258753

[B45] Chou J. Chen J. J. Gross M. Roizman B. (1995). Association of a M(r) 90,000 phosphoprotein with protein kinase PKR in cells exhibiting enhanced phosphorylation of translation initiation factor eIF-2 α and premature shutoff of protein synthesis after infection with γ 134.5-mutants of herpes simplex virus 1. Proc. Natl. Acad. Sci. U S A 92, 10516–10520. 10.1073/pnas.92.23.10516 7479831 PMC40642

[B46] Christen-Zaech S. Kraftsik R. Pillevuit O. Kiraly M. Martins R. Khalili K.. (2003). Early olfactory involvement in Alzheimer’s disease. Can. J. Neurol. Sci. 30, 20–25. 10.1017/S0317167100002389 12619779

[B47] Civitelli L. Marcocci M. E. Celestino I. Piacentini R. Garaci E. Grassi C.. (2015). Herpes simplex virus type 1 infection in neurons leads to production and nuclear localization of APP intracellular domain (AICD): implications for Alzheimer’s disease pathogenesis. J. Neurovirol. 21, 480–490. 10.1007/s13365-015-0344-0 25925093

[B48] Conrady C. D. Drevets D. A. Carr D. J. (2010). Herpes simplex type I (HSV-1) infection of the nervous system: is an immune response a good thing? J. Neuroimmunol. 220, 1–9. 10.1016/j.jneuroim.2009.09.013 19819030 PMC2835842

[B49] Corder E. H. Robertson K. Lannfelt L. Bogdanovic N. Eggertsen G. Wilkins J.. (1998). HIV-infected subjects with the E4 allele for APOE have excess dementia and peripheral neuropathy. Nat. Med. 4, 1182–1184. 10.1038/2677 9771753

[B50] Cribbs D. H. Azizeh B. Y. Cotman C. W. LaFerla F. M. (2000). Fibril formation and neurotoxicity by a herpes simplex virus glycoprotein B fragment with homology to the Alzheimer’s Aβ peptide. Biochemistry 39, 5988–5994. 10.1021/bi000029f 10821670

[B51] D’Aiuto L. Prasad K. M. Upton C. H. Viggiano L. Milosevic J. Raimondi G.. (2015). Persistent infection by HSV-1 is associated with changes in functional architecture of iPSC derived neurons and brain activation patterns underlying working memory performance. Schizophr. Bull. 41, 123–132. 10.1093/schbul/sbu032 24622295 PMC4266288

[B52] De Chiara G. Marcocci M. E. Civitelli L. Argnani R. Piacentini R. Ripoli C.. (2010). APP processing induced by herpes simplex virus type 1 (HSV-1) yields several APP fragments in human and rat neuronal cells. PLoS One 5:e13989. 10.1371/journal.pone.0013989 21085580 PMC2981559

[B53] Debatin L. Streffer J. Geissen M. Matschke J. Aguzzi A. Glatzel M. (2008). Association between deposition of β-amyloid and pathological prion protein in sporadic Creutzfeldt-Jakob disease. Neurodegener. Dis. 5, 347–354. 10.1159/000121389 18349519

[B54] De-Paula V. J. Radanovic M. Diniz B. S. Forlenza O. V. (2012). Alzheimer’s disease. Subcell. Biochem. 65, 329–352. 10.1007/978-94-007-5416-4_14 23225010

[B56] Dickerson F. B. Boronow J. J. Stallings C. Origoni A. E. Ruslanova I. Yolken R. H. (2003). Association of serum antibodies to herpes simplex virus 1 with cognitive deficits in individuals with schizophrenia. Arch. Gen. Psychiatry 60, 466–472. 10.1001/archpsyc.60.5.466 12742867

[B55] Dickerson F. Stallings C. Origoni A. Vaughan C. Khushalani S. Yolken R. (2012). Additive effects of elevated C-reactive protein and exposure to herpes simplex virus type 1 on cognitive impairment in individuals with schizophrenia. Schizophr. Res. 134, 83–88. 10.1016/j.schres.2011.10.003 22048011

[B57] Egan K. P. Wu S. Wigdahl B. Jennings S. R. (2013). Immunological control of herpes simplex virus infections. J. Neurovirol. 19, 328–345. 10.1007/s13365-013-0189-3 23943467 PMC3758505

[B58] Elion G. B. (1982). Mechanism of action and selectivity of acyclovir. Am. J. Med. 73, 7–13. 10.1016/0002-9343(82)90055-9 6285736

[B59] Emilsson L. Saetre P. Jazin E. (2006). Alzheimer’s disease: mRNA expression profiles of multiple patients show alterations of genes involved with calcium signaling. Neurobiol. Dis. 21, 618–625. 10.1016/j.nbd.2005.09.004 16257224

[B60] Esiri M. M. Biddolph S. C. Morris C. S. (1998). Prevalence of Alzheimer plaques in AIDS. J. Neurol. Neurosurg. Psychiatry 65, 29–33. 10.1136/jnnp.65.1.29 9667557 PMC2170157

[B61] Feldman L. T. Ellison A. R. Voytek C. C. Yang L. Krause P. Margolis T. P. (2002). Spontaneous molecular reactivation of herpes simplex virus type 1 latency in mice. Proc. Natl. Acad. Sci. U S A 99, 978–983. 10.1073/pnas.022301899 11773630 PMC117416

[B62] Friedman J. E. Zabriskie J. B. Plank C. Ablashi D. Whitman J. Shahan B. (2005). A randomized clinical trial of valacyclovir in multiple sclerosis. Mult. Scler. 11, 286–295. 10.1191/1352458505ms1185oa 15957509

[B63] Fruchter E. Goldberg S. Fenchel D. Grotto I. Ginat K. Weiser M. (2015). The impact of Herpes simplex virus type 1 on cognitive impairments in young, healthy individuals-A historical prospective study. Schizophr. Res. 168, 292–296. 10.1016/j.schres.2015.08.036 26362735

[B65] Gale S. D. Erickson L. D. Berrett A. Brown B. L. Hedges D. W. (2016). Infectious disease burden and cognitive function in young to middle-aged adults. Brain Behav. Immun. 52, 161–168. 10.1016/j.bbi.2015.10.014 26598104

[B64] Gale S. C. Gao L. Mikacenic C. Coyle S. M. Rafaels N. Murray Dudenkov T.. (2014). APOε4 is associated with enhanced *in vivo* innate immune responses in human subjects. J. Allergy Clin. Immunol. 134, 127–134. 10.1016/j.jaci.2014.01.032 24655576 PMC4125509

[B66] Galvan V. Roizman B. (1998). Herpes simplex virus 1 induces and blocks apoptosis at multiple steps during infection and protects cells from exogenous inducers in a cell-type-dependent manner. Proc. Natl. Acad. Sci. U S A 95, 3931–3936. 10.1073/pnas.95.7.3931 9520470 PMC19940

[B67] Gao H. M. Hong J. S. (2008). Why neurodegenerative diseases are progressive: uncontrolled inflammation drives disease progression. Trends Immunol. 29, 357–365. 10.1016/j.it.2008.05.002 18599350 PMC4794280

[B68] Gérard H. C. Dreses-Werringloer U. Wildt K. S. Deka S. Oszust C. Balin B. J.. (2006). Chlamydophila (Chlamydia) pneumoniae in the Alzheimer’s brain. FEMS Immunol. Med. Microbiol. 48, 355–366. 10.1111/j.1574-695X.2006.00154.x 17052268

[B69] Goldberg L. H. Kaufman R. Kurtz T. O. Conant M. A. Eron L. J. Batenhorst R. L.. (1993). Long-term suppression of recurrent genital herpes with acyclovir. A 5-year benchmark. Acyclovir Study Group. Arch. Dermatol. 129, 582–587. 10.1001/archderm.129.5.582 8481018

[B70] Green D. A. Masliah E. Vinters H. V. Beizai P. Moore D. J. Achim C. L. (2005). Brain deposition of β-amyloid is a common pathologic feature in HIV positive patients. AIDS 19, 407–411. 10.1097/01.aids.0000161770.06158.5c 15750394

[B71] Griffin W. S. (2013). Neuroinflammatory cytokine signaling and Alzheimer’s disease. N. Engl. J. Med. 368, 770–771. 10.1056/NEJMcibr1214546 23425171

[B72] Griffin W. S. Sheng J. G. Royston M. C. Gentleman S. M. McKenzie J. E. Graham D. I.. (1998). Glial-neuronal interactions in Alzheimer’s disease: the potential role of a ‘cytokine cycle’ in disease progression. Brain Pathol. 8, 65–72. 10.1111/j.1750-3639.1998.tb00136.x 9458167 PMC8098321

[B73] Guzman-Sanchez F. Valdivieso F. Burgos J. S. (2012). Aging-related neurostructural, neuropathological and behavioral changes associated with herpes simplex virus type 1 brain infection in mice. J. Alzheimers Dis. 30, 779–790. 10.3233/JAD-2012-120070 22466000

[B74] Halford W. P. Gebhardt B. M. Carr D. J. (1996). Persistent cytokine expression in trigeminal ganglion latently infected with herpes simplex virus type 1. J. Immunol. 157, 3542–3549. 8871654

[B75] Halford W. P. Gebhardt B. M. Carr D. J. (1997). Acyclovir blocks cytokine gene expression in trigeminal ganglia latently infected with herpes simplex virus type 1. Virology 238, 53–63. 10.1006/viro.1997.8806 9375008

[B76] Harris S. A. Harris E. A. (2015). Herpes simplex virus type 1 and other pathogens are key causative factors in sporadic Alzheimer’s disease. J. Alzheimers Dis. 9, 319–353. 10.3233/JAD-142853 26401998 PMC4923765

[B77] Held K. Derfuss T. (2011). Control of HSV-1 latency in human trigeminal ganglia—current overview. J. Neurovirol. 17, 518–527. 10.1007/s13365-011-0063-0 22139603

[B78] Hemling N. Röyttä M. Rinne J. Pöllänen P. Broberg E. Tapio V.. (2003). Herpesviruses in brains in Alzheimer’s and Parkinson’s diseases. Ann. Neurol. 54, 267–271. 10.1002/ana.10662 12891684

[B79] Hirai K. Aliev G. Nunomura A. Fujioka H. Russell R. L. Atwood C. S.. (2001). Mitochondrial abnormalities in Alzheimer’s disease. J. Neurosci. 21, 3017–3023. 11312286 10.1523/JNEUROSCI.21-09-03017.2001 PMC6762571

[B80] Ho G. J. Drego R. Hakimian E. Masliah E. (2005). Mechanisms of cell signaling and inflammation in Alzheimer’s disease. Curr. Drug Targets Inflamm. Allergy 4, 247–256. 10.2174/1568010053586237 15853747

[B82] Holtzman D. M. Herz J. Bu G. (2012). Apolipoprotein E and apolipoprotein E receptors: normal biology and roles in Alzheimer disease. Cold Spring Harb. Perspect. Med. 2:a006312. 10.1101/cshperspect.a006312 22393530 PMC3282491

[B83] Hooper C. Killick R. Lovestone S. (2008). The GSK3 hypothesis of Alzheimer’s disease. J. Neurochem. 104, 1433–1439. 10.1111/j.1471-4159.2007.05194.x 18088381 PMC3073119

[B84] Ill-Raga G. Palomer E. Wozniak M. A. Ramos-Fernández E. Bosch-Morató M. Tajes M.. (2011). Activation of PKR causes amyloid ß-peptide accumulation via de-repression of BACE1 expression. PLoS One 6:e21456. 10.1371/journal.pone.0021456 21738672 PMC3125189

[B85] Itabashi S. Arai H. Matsui T. Higuchi S. Sasaki H. (1997). Herpes simplex virus and risk of Alzheimer’s disease. Lancet 349:1102. 10.1016/S0140-6736(05)62325-2 9107270

[B93] Itzhaki R. F. (2011). Herpes Simplex and Alzheimer’s-Time to Think Again? Alzforum Available online at: http://www.alzforum.org/webinars/herpes-simplex-and-alzheimers-time-think-again. Accessed on June 27, 2017.

[B90] Itzhaki R. F. (2014). Herpes simplex virus type 1 and Alzheimer’s disease: increasing evidence for a major role of the virus. Front. Aging Neurosci. 6:202. 10.3389/fnagi.2014.00202 25157230 PMC4128394

[B91] Itzhaki R. F. (2016). Herpes and Alzheimer’s disease: subversion in the central nervous system and how it might be halted. J. Alzheimers Dis. 54, 1273–1281. 10.3233/jad-160607 27497484

[B92] Itzhaki R. F. (2017). Herpes simplex virus type 1 and Alzheimer’s disease: possible mechanisms and signposts. FASEB J. 31, 3216–3226. 10.1096/fj.201700360 28765170

[B86] Itzhaki R. F. Cosby S. L. Wozniak M. A. (2008). Herpes simplex virus type 1 and Alzheimer’s disease: the autophagy connection. J. Neurovirol. 14, 1–4. 10.1080/13550280701802543 18300070

[B94] Itzhaki R. F. Lathe R. Balin B. J. Ball M. J. Bearer E. L. Braak H.. (2016). Microbes and Alzheimer’s disease. J. Alzheimers Dis. 51, 979–984. 10.3233/JAD-160152 26967229 PMC5457904

[B87] Itzhaki R. F. Lin W. R. Shang D. Wilcock G. K. Faragher B. Jamieson G. A. (1997). Herpes simplex virus type 1 in brain and risk of Alzheimer’s disease. Lancet 349, 241–244. 10.1016/S0140-6736(96)10149-5 9014911

[B88] Itzhaki R. F. Wozniak M. A. (2006). Herpes simplex virus type 1, apolipoprotein E, and cholesterol: a dangerous liaison in Alzheimer’s disease and other disorders. Prog. Lipid Res. 45, 73–90. 10.1016/j.plipres.2005.11.003 16406033

[B89] Itzhaki R. F. Wozniak M. A. (2008). Herpes simplex virus type 1 in Alzheimer’s disease: the enemy within. J. Alzheimers Dis. 13, 393–405. 10.3233/jad-2008-13405 18487848

[B95] Iwata N. Higuchi M. Saido T. C. (2005). Metabolism of amyloid-β peptide and Alzheimer’s disease. Pharmacol. Ther. 108, 129–148. 10.1016/j.pharmthera.2005.03.010 16112736

[B96] Izadpanah A. Gallo R. L. (2005). Antimicrobial peptides. J. Am. Acad. Dermatol. 52, 381–390; quiz 391–392. 10.1016/j.jaad.2004.08.026 15761415

[B97] Jamieson G. A. Maitland N. J. Wilcock G. K. Craske J. Itzhaki R. F. (1991). Latent herpes simplex virus type 1 in normal and Alzheimer’s disease brains. J. Med. Virol. 33, 224–227. 10.1002/jmv.1890330403 1649907

[B98] Jamieson G. A. Maitland N. J. Wilcock G. K. Yates C. M. Itzhaki R. F. (1992). Herpes simplex virus type 1 DNA is present in specific regions of brain from aged people with and without senile dementia of the Alzheimer type. J. Pathol. 167, 365–368. 10.1002/path.1711670403 1328575

[B99] Kagan B. L. Jang H. Capone R. Teran Arce F. Ramachandran S. Lal R.. (2012). Antimicrobial properties of amyloid peptides. Mol. Pharm. 9, 708–717. 10.1021/mp200419b 22081976 PMC3297685

[B100] Kamer A. R. Craig R. G. Pirraglia E. Dasanayake A. P. Norman R. G. Boylan R. J. (2009). TNF-α and antibodies to periodontal bacteria discriminate between Alzheimer’s disease patients and normal subjects. J. Neuroimmunol. 216, 92–97. 10.1016/j.jneuroim.2009.08.013 19767111 PMC2783848

[B101] Katan M. Moon Y. P. Paik M. C. Sacco R. L. Wright C. B. Elkind M. S. (2013). Infectious burden and cognitive function: the northern manhattan study. Neurology 80, 1209–1215. 10.1212/WNL.0b013e3182896e79 23530151 PMC3691781

[B102] Kaufman H. E. Azcuy A. M. Varnell E. D. Sloop G. D. Thompson H. W. Hill J. M. (2005). HSV-1 DNA in tears and saliva of normal adults. Invest. Ophthalmol. Vis. Sci. 46, 241–247. 10.1167/iovs.04-0614 15623779 PMC1200985

[B103] Kaye S. Choudhary A. (2006). Herpes simplex keratitis. Prog. Retin. Eye Res. 25, 355–380. 10.1016/j.preteyeres.2006.05.001 16807055

[B104] Klapper P. E. Cleator G. M. Longson M. (1984). Mild forms of herpes encephalitis. J. Neurol. Neurosurg. Psychiatry 47, 1247–1250. 10.1136/jnnp.47.11.1247 6094737 PMC1028097

[B105] Kobayashi N. Nagata T. Shinagawa S. Oka N. Shimada K. Shimizu A.. (2013). Increase in the IgG avidity index due to herpes simplex virus type 1 reactivation and its relationship with cognitive function in amnestic mild cognitive impairment and Alzheimer’s disease. Biochem. Biophys. Res. Commun. 430, 907–911. 10.1016/j.bbrc.2012.12.054 23261465

[B106] Koch S. Solana R. Dela Rosa O. Pawelec G. (2006). Human cytomegalovirus infection and T cell immunosenescence: a mini review. Mech. Ageing Dev. 127, 538–543. 10.1016/j.mad.2006.01.011 16513159

[B107] Koelle D. M. Magaret A. Warren T. Schellenberg G. D. Wald A. (2010). APOE genotype is associated with oral herpetic lesions but not genital or oral herpes simplex virus shedding. Sex. Transm. Infect. 86, 202–206. 10.1136/sti.2009.039735 20410080 PMC2881187

[B108] Kolarova M. García-Sierra F. Bartos A. Ricny J. Ripova D. (2012). Structure and pathology of tau protein in Alzheimer disease. Int. J. Alzheimers Dis. 2012:731526. 10.1155/2012/731526 22690349 PMC3368361

[B109] Korr G. Thamm M. Czogiel I. Poethko-Mueller C. Bremer V. Jansen K. (2017). Decreasing seroprevalence of herpes simplex virus type 1 and type 2 in Germany leaves many people susceptible to genital infection: time to raise awareness and enhance control. BMC Infect. Dis. 17:471. 10.1186/s12879-017-2527-1 28683784 PMC5500947

[B110] Kountouras J. Boziki M. Gavalas E. Zavos C. Deretzi G. Grigoriadis N.. (2009). Increased cerebrospinal fluid Helicobacter pylori antibody in Alzheimer’s disease. Int. J. Neurosci. 119, 765–777. 10.1080/00207450902782083 19326283

[B111] Kovács T. Cairns N. J. Lantos P. L. (2001). Olfactory centres in Alzheimer’s disease: olfactory bulb is involved in early Braak’s stages. Neuroreport 12, 285–288. 10.1097/00001756-200102120-00021 11209936

[B112] Kramer T. Enquist L. W. (2012). αherpesvirus infection disrupts mitochondrial transport in neurons. Cell Host Microbe 11, 504–514. 10.1016/j.chom.2012.03.005 22607803 PMC3358700

[B113] Kuhlmann I. Minihane A. M. Huebbe P. Nebel A. Rimbach G. (2010). Apolipoprotein E genotype and hepatitis C, HIV and herpes simplex disease risk: a literature review. Lipids Health Dis. 9:8. 10.1186/1476-511x-9-8 20109174 PMC2830997

[B114] Kukhanova M. K. Korovina A. N. Kochetkov S. N. (2014). Human herpes simplex virus: life cycle and development of inhibitors. Biochemistry (Mosc) 79, 1635–1652. 10.1134/s0006297914130124 25749169

[B115] Kumar D. K. Choi S. H. Washicosky K. J. Eimer W. A. Tucker S. Ghofrani J.. (2016). Amyloid-β peptide protects against microbial infection in mouse and worm models of Alzheimer’s disease. Sci. Transl. Med. 8:340ra72. 10.1126/scitranslmed.aaf1059 27225182 PMC5505565

[B116] Kumar P. Kumar D. Jha S. K. Jha N. K. Ambasta R. K. (2016). Ion channels in neurological disorders. Adv. Protein Chem. Struct. Biol. 103, 97–136. 10.1016/bs.apcsb.2015.10.006 26920688

[B118] Lambert J. C. Heath S. Even G. Campion D. Sleegers K. Hiltunen M.. (2009). Genome-wide association study identifies variants at CLU and CR1 associated with Alzheimer’s disease. Nat. Genet. 41, 1094–1099. 10.1038/ng.439 19734903

[B119] Lambert J. C. Zelenika D. Hiltunen M. Chouraki V. Combarros O. Bullido M. J.. (2011). Evidence of the association of BIN1 and PICALM with the AD risk in contrasting European populations. Neurobiol. Aging 32, 756.e11–756.e15. 10.1016/j.neurobiolaging.2010.11.022 21220176

[B120] Lassmann H. Weiler R. Fischer P. Bancher C. Jellinger K. Floor E.. (1992). Synaptic pathology in Alzheimer’s disease: immunological data for markers of synaptic and large dense-core vesicles. Neuroscience 146, 1–8. 10.1016/0306-4522(92)90003-k 1594095

[B121] Lathe R. Haas J. G. (2017). Distribution of cellular HSV-1 receptor expression in human brain. J. Neurovirol. 23, 376–384. 10.1007/s13365-016-0504-x 27981441 PMC5440480

[B122] Lathe R. Sapronova A. Kotelevtsev Y. (2014). Atherosclerosis and Alzheimer—diseases with a common cause? Inflammation, oxysterols, vasculature. BMC Geriatr. 14:36. 10.1186/1471-2318-14-36 24656052 PMC3994432

[B123] Letenneur L. Pérès K. Fleury H. Garrigue I. Barberger-Gateau P. Helmer C.. (2008). Seropositivity to herpes simplex virus antibodies and risk of Alzheimer’s disease: a populationbased cohort study. PLoS One 3:e3637. 10.1371/journal.pone.0003637 18982063 PMC2572852

[B124] Lewerenz J. Maher P. (2015). Chronic glutamate toxicity in neurodegenerative diseases-what is the evidence? Front. Neurosci. 9:469. 10.3389/fnins.2015.00469 26733784 PMC4679930

[B125] Li L. Li Z. Li X. Wang E. Lang F. Xia Y.. (2016). Reactivation of HSV-1 following explant of tree shrew brain. J. Neurovirol. 22, 293–306. 10.1007/s13365-015-0393-4 26501779 PMC4899501

[B126] Liang Z. Liu F. Grundke-Iqbal I. Iqbal K. Gong C. X. (2007). Down-regulation of cAMP-dependent protein kinase by over-activated calpain in Alzheimer disease brain. J. Neurochem. 103, 2462–2470. 10.1111/j.1471-4159.2007.04942.x 17908236 PMC2262109

[B127] Liberski P. P. (1994). Transmissible cerebral amyloidoses as a model for Alzheimer’s disease. An ultrastructural perspective. Mol. Neurobiol. 8, 67–77. 10.1007/bf02778009 7522013

[B128] Licastro F. Carbone I. Ianni M. Porcellini E. (2011). Gene signature in Alzheimer’s disease and environmental factors: the virus chronicle. J. Alzheimers Dis. 27, 809–817. 10.3233/JAD-2011-110755 21891868

[B129] Limongi D. Baldelli S. (2016). Redox imbalance and viral infections in neurodegenerative diseases. Oxid. Med. Cell. Longev. 2016:6547248. 10.1155/2016/6547248 27110325 PMC4826696

[B131] Lin W. R. Graham J. MacGowan S. M. Wilcock G. K. Itzhaki R. F. (1998). Alzheimer’s disease, herpes virus in brain, apolipoprotein E4 and herpes labialis. Alzheimers Rep. 1, 173–178.

[B130] Lin W. R. Jennings R. Smith T. L. Wozniak M. A. Itzhaki R. F. (2001). Vaccination prevents latent HSV1 infection of mouse brain. Neurobiol. Aging 22, 699–703. 10.1016/s0197-4580(01)00268-8 11705626

[B132] Little C. S. Hammond C. J. MacIntyre A. Balin B. J. Appelt D. M. (2004). Chlamydia pneumoniae induces Alzheimer-like amyloid plaques in brains of BALB/c mice. Neurobiol. Aging 25, 419–429. 10.1016/s0197-4580(03)00127-1 15013562

[B133] Liu S. Y. Aliyari R. Chikere K. Li G. Marsden M. D. Smith J. K.. (2013). Interferon-inducible cholesterol-25-hydroxylase broadly inhibits viral entry by production of 25-hydroxycholesterol. Immunity 38, 92–105. 10.1016/j.immuni.2012.11.005 23273844 PMC3698975

[B134] Lokensgard J. R. Hu S. Sheng W. vanOijen M. Cox D. Cheeran M. C.. (2001). Robust expression of TNF-α, IL-1β, RANTES, and IP-10 by human microglial cells during nonproductive infection with herpes simplex virus. J. Neurovirol. 7, 208–219. 10.1080/13550280152403254 11517395

[B135] Looker K. J. Magaret A. S. May M. T. Turner K. M. Vickerman P. Gottlieb S. L.. (2015). Global and regional estimates of prevalent and incident herpes simplex virus type 1 infections in 2012. PLoS One 10:e0140765. 10.1371/journal.pone.0140765 26510007 PMC4624804

[B136] Lopez J. R. Lyckman A. Oddo S. Laferla F. M. Querfurth H. W. Shtifman A. (2008). Increased intraneuronal resting [Ca^2+^] in adult Alzheimer’s disease mice. J. Neurochem. 105, 262–271. 10.1111/j.1471-4159.2007.05135.x 18021291

[B117] Lövheim H. Gilthorpe J. Adolfsson R. Nilsson L. G. Elgh F. (2015a). Reactivated herpes simplex infection increases the risk of Alzheimer’s disease. Alzheimers Dement. 11, 593–599. 10.1016/j.jalz.2014.04.522 25043910

[B137] Lövheim H. Gilthorpe J. Johansson A. Eriksson S. Hallmans G. Elgh F. (2015b). Herpes simplex infection and the risk of Alzheimer’s disease-A nested case-control study. Alzheimers Dement. 11, 587–592. 10.1016/j.jalz.2014.07.157 25304990

[B138] Lue L. F. Walker D. G. Rogers J. (2001). Modeling microglial activation in Alzheimer’s disease with human postmortem microglial cultures. Neurobiol. Aging 22, 945–956. 10.1016/s0197-4580(01)00311-6 11755003

[B139] Lukiw W. J. Cui J. G. Yuan L. Y. Bhattacharjee P. S. Corkern M. Clement C.. (2010). Acyclovir or Aβ42 peptides attenuate HSV-1-induced miRNA-146a levels in human primary brain cells. Neuroreport 21, 922–927. 10.1097/WNR.0b013e32833da51a 20683212 PMC2953363

[B140] Lurain N. S. Hanson B. A. Martinson J. Leurgans S. E. Landay A. L. Bennett D. A.. (2013). Virological and immunological characteristics of human cytomegalovirus infection associated with Alzheimer disease. J. Infect. Dis. 208, 564–572. 10.1093/infdis/jit210 23661800 PMC3719902

[B141] Lycke J. Malmeström C. Ståhle L. (2003). Acyclovir levels in serum and cerebrospinal fluid after oral administration of valacyclovir. Antimicrob. Agents Chemother. 47, 2438–2441. 10.1128/aac.47.8.2438-2441.2003 12878501 PMC166099

[B142] Lynch M. A. (2014). The impact of neuroimmune changes on development of amyloid pathology; relevance to Alzheimer’s disease. Immunology 141, 292–301. 10.1111/imm.12156 23876085 PMC3930368

[B143] Mahley R. W. Rall S. C. Jr. (2000). Apolipoprotein E: far more than a lipid transport protein. Annu. Rev. Genomics Hum. Genet. 1, 507–537. 10.1146/annurev.genom.1.1.507 11701639

[B144] Mancuso R. Baglio F. Cabinio M. Calabrese E. Hernis A. Nemni R.. (2014). Titers of herpes simplex virus type 1 antibodies positively correlate with grey matter volumes in Alzheimer’s disease. J. Alzheimers Dis. 38, 741–745. 10.3233/JAD-130977 24072067

[B145] Manoharan S. Guillemin G. J. Abiramasundari R. S. Essa M. M. Akbar M. Akbar M. D. (2016). The role of reactive oxygen species in the pathogenesis of Alzheimer’s disease, Parkinson’s disease, and Huntington’s disease: a mini review. Oxid. Med. Cell. Longev. 2016:8590578. 10.1155/2016/8590578 28116038 PMC5223034

[B146] Marchi S. Trombetta C. M. Gasparini R. Temperton N. Montomoli E. (2017). Epidemiology of herpes simplex virus type 1 and 2 in Italy: a seroprevalence study from 2000 to 2014. J. Prev. Med. Hyg. 58, E27–E33. 28515628 PMC5432775

[B147] Margolis T. P. Elfman F. L. Leib D. Pakpour N. Apakupakul K. Imai Y.. (2007). Spontaneous reactivation of herpes simplex virus type 1 in latently infected murine sensory ganglia. J. Virol. 81, 11069–11074. 10.1128/jvi.00243-07 17686862 PMC2045564

[B148] Marques A. R. Straus S. E. Fahle G. Weir S. Csako G. Fischer S. H. (2001). Lack of association betweenHSV-1DNA in the brain, Alzheimer’s disease and apolipoprotein E4. J. Neurovirol. 7, 82–83. 10.1080/135502801300069773 11519487

[B149] Marr R. A. Guan H. Rockenstein E. Kindy M. Gage F. H. Verma I.. (2004). Neprilysin regulates amyloid β peptide levels. J. Mol. Neurosci. 22, 5–11. 10.1385/jmn:22:1-2:5 14742905

[B150] Martin C. Aguila B. Araya P. Vio K. Valdivia S. Zambrano A.. (2014). Inflammatory and neurodegeneration markers during asymptomatic HSV-1 reactivation. J. Alzheimers Dis. 39, 849–859. 10.3233/JAD-131706 24296813

[B151] Martinez-Diaz G. J. Hsia R. (2011). Altered mental status from acyclovir. J. Emerg. Med. 41, 55–58. 10.1016/j.jemermed.2009.08.048 19926428

[B152] Marton R. Gotlieb-Stematsky T. Klein C. Lahat E. Arlazoroff A. (1995). Mild form of acute herpes simplex encephalitis in childhood. Brain Dev. 17, 360–361. 10.1016/0387-7604(95)00075-m 8579225

[B153] Masliah E. Mallory M. Alford M. DeTeresa R. Hansen L. A. McKeel D. W. Jr.. (2001). Altered expression of synaptic proteins occurs early during progression of Alzheimer’s disease. Neurology 56, 127–129. 10.1212/WNL.56.1.127 11148253

[B154] Matsuzaki K. Yamakuni T. Hashimoto M. Haque A. M. Shido O. Mimaki Y.. (2006). Nobiletin restoring β-amyloid-impaired CREB phosphorylation rescues memory deterioration in Alzheimer’s disease model rats. Neurosci. Lett. 400, 230–234. 10.1016/j.neulet.2006.02.077 16581185

[B155] Mayeux R. Stern Y. (2012). Epidemiology of Alzheimer disease. Cold Spring Harb. Perspect. Med. 2:a006239. 10.1101/cshperspect.a006239 22908189 PMC3405821

[B156] McGeer P. L. Akiyama H. Itagaki S. McGeer E. G. (1989). Activation of the classical complement pathway in brain tissue of Alzheimer patients. Neurosci. Lett. 107, 341–346. 10.1016/0304-3940(89)90843-4 2559373

[B157] McKhann G. M. Knopman D. S. Chertkow H. Hyman B. T. Jack C. R. Jr. Kawas C. H.. (2011). The diagnosis of dementia due to Alzheimer’s disease: recommendations from the National Institute on Aging-Alzheimer’s Association workgroups on diagnostic guidelines for Alzheimer’s disease. Alzheimers Dement. 7, 263–269. 10.1016/j.jalz.2011.03.005 21514250 PMC3312024

[B158] McLean J. H. Shipley M. T. Bernstein D. I. Corbett D. (1993). Selective lesions of neural pathways following viral inoculation of the olfactory bulb. Exp. Neurol. 122, 209–222. 10.1006/exnr.1993.1121 8104818

[B159] McQuaid S. Allen I. V. McMahon J. Kirk J. (1994). Association of measles virus with neurofibrillary tangles in subacute sclerosing panencephalitis: a combined *in situ* hybridization and immunocytochemical investigation. Neuropathol. Appl. Neurobiol 20, 103–110. 10.1111/j.1365-2990.1994.tb01168.x 8072641

[B160] Meyding-Lamadé U. Haas J. Lamadé W. Stingele K. Kehm R. Fäth A.. (1998). Herpes simplex virus encephalitis: long-term comparative study of viral load and the expression of immunologic nitric oxide synthase in mouse brain tissue. Neurosci. Lett. 244, 9–12. 10.1016/s0304-3940(98)00115-3 9578132

[B161] Michael B. D. Griffiths M. J. Granerod J. Brown D. Keir G. Wnek G.. (2016). The interleukin-1 balance during encephalitis is associated with clinical severity, blood-brain barrier permeability, neuroimaging changes, and disease outcome. J. Infect. Dis. 213, 1651–1660. 10.1093/infdis/jiv771 26712949 PMC4837908

[B162] Miklossy J. (1998). Chronic inflammation and amyloidogenesis in Alzheimer’s disease: putative role of bacterial peptidoglycan, a potent inflammatory and amyloidogenic factor. Alzheimers Rev. 3, 45–51.

[B167] Miklossy J. (2015). Historic evidence to support a causal relationship between spirochetal infections and Alzheimer’s disease. Front. Aging Neurosci. 7:46. 10.3389/fnagi.2015.00046 25932012 PMC4399390

[B169] Miklossy J. (2011a). Emerging roles of pathogens in Alzheimer disease. Expert Rev. Mol. Med. 13:e30. 10.1017/S1462399411002006 21933454

[B168] Miklossy J. (2011b). Alzheimer’s disease—a neurospirochetosis. Analysis of the evidence following Koch’s and Hill’s criteria. J. Neuroinflammation 8:90. 10.1186/1742-2094-8-90 21816039 PMC3171359

[B163] Miklossy J. Darekar P. Gern L. Janzer R. C. Bosman F. T. (1996). Bacterial peptidoglycan in neuritic plaque in Alzheimer’s disease. Alzheimers Res. 2, 95–100.

[B164] Miklossy J. Khalili K. Gern L. Ericson R. L. Darekar P. Bolle L.. (2004). Borrelia burgdorferi persists in the brain in chronic lyme neuroborreliosis and may be associated with Alzheimer disease. J. Alzheimers Dis. 6, 639–649; discussion 673–681. 10.3233/jad-2004-6608 15665404

[B165] Miklossy J. Kis A. Radenovic A. Miller L. Forro L. Martins R.. (2006a). β-amyloid deposition and Alzheimer’s type changes induced by Borrelia spirochetes. Neurobiol. Aging 27, 228–236. 10.1016/j.neurobiolaging.2005.01.018 15894409

[B166] Miklossy J. Rosemberg S. McGeer P. L. (2006b). “β amyloid deposition in the atrophic form of general paresis. Alzheimer’s disease: new advances,” in Proceedings of the 10th International Congress on Alzheimer’s Disease (ICAD), eds Iqbal K. Winblad B. Avila J. (Bologna, Italy: Medimond, International Proceedings), 429–433.

[B170] Miyazawa K. Kipkorir T. Tittman S. Manuelidis L. (2012). Continuous production of prions after infectious particles are eliminated: implications for Alzheimer’s disease. PLoS One 7:e35471. 10.1371/journal.pone.0035471 22509412 PMC3324552

[B171] Mocchetti I. Bachis A. Esposito G. Turner S. R. Taraballi F. Tasciotti E.. (2014). Human immunodeficiency virus-associated dementia: a link between accumulation of viral proteins and neuronal degeneration. Curr. Trends Neurol. 8, 71–85. 26069421 PMC4461001

[B172] Mondragón-Rodríguez S. Perry G. Zhu X. Moreira P. I. Acevedo-Aquino M. C. Williams S. (2013). Phosphorylation of tau protein as the link between oxidative stress, mitochondrial dysfunction, and connectivity failure: implications for Alzheimer’s disease. Oxid. Med. Cell. Longev. 2013:940603. 10.1155/2013/940603 23936615 PMC3723250

[B173] Montagne A. Barnes S. R. Sweeney M. D. Halliday M. R. Sagare A. P. Zhao Z.. (2015). Blood-brain barrier breakdown in the aging human hippocampus. Neuron 85, 296–302. 10.1016/j.neuron.2014.12.032 25611508 PMC4350773

[B174] Mori I. Kimura Y. Naiki H. Matsubara R. Takeuchi T. Yokochi T.. (2004). Reactivation of HSV-1 in the brain of patients with familial Alzheimer’s disease. J. Med. Virol. 73, 605–611. 10.1002/jmv.20133 15221907

[B175] Mori I. Nishiyama Y. Yokochi T. Kimura Y. (2005). Olfactory transmission of neurotropic viruses. J. Neurovirol. 11, 129–137. 10.1080/13550280590922793 16036791

[B176] Naj A. C. Schellenberg G. D. Alzheimer’s Disease Genetics Consortium (ADGC) (2017). Genomic variants, genes, and pathways of Alzheimer’s disease: an overview. Am. J. Med. Genet. B Neuropsychiatr. Genet. 174, 5–26. 10.1002/ajmg.b.32499 27943641 PMC6179157

[B177] Nasrallah G. K. Dargham S. R. Mohammed L. I. Abu-Raddad L. J. (2018). Estimating seroprevalence of herpes simplex virus type 1 among different Middle East and North African male populations residing in Qatar. J. Med. Virol. 90, 184–190. 10.1002/jmv.24916 28817197 PMC5724503

[B178] Nicoll M. P. Proença J. T. Efstathiou S. (2012). The molecular basis of herpes simplex virus latency. FEMS Microbiol. Rev. 36, 684–705. 10.1111/j.1574-6976.2011.00320.x 22150699 PMC3492847

[B179] Nixon R. A. (2007). Autophagy, amyloidogenesis and Alzheimer disease. J. Cell Sci. 120, 4081–4091. 10.1242/jcs.019265 18032783

[B180] Nixon R. A. Yang D. S. (2011). Autophagy failure in Alzheimer’s disease—locating the primary defect. Neurobiol. Dis. 43, 38–45. 10.1016/j.nbd.2011.01.021 21296668 PMC3096679

[B181] Nucci C. Palamara A. T. Ciriolo M. R. Nencioni L. Savini P. D’Agostini C.. (2000). Imbalance in corneal redox state during herpes simplex virus 1-induced keratitis in rabbits. Effectiveness of exogenous glutathione supply. Exp. Eye Res. 70, 215–220. 10.1006/exer.1999.0782 10655147

[B182] O’Connell D. Liang C. (2016). Autophagy interaction with herpes simplex virus type-1 infection. Autophagy 12, 451–459. 10.1080/15548627.2016.1139262 26934628 PMC4836034

[B183] Ohnishi S. Koide A. Koide S. (2000). Solution conformation and amyloid-like fibril formation of a polar peptide derived from a β-hairpin in the OspA single-layer β-sheet. J. Mol. Biol. 301, 477–489. 10.1006/jmbi.2000.3980 10926522

[B184] Orvedahl A. Alexander D. Tallóczy Z. Sun Q. Wei Y. Zhang W.. (2007). HSV-1 ICP34.5 confers neurovirulence by targeting the Beclin 1 autophagy protein. Cell Host Microbe 1, 23–35. 10.1016/j.chom.2006.12.001 18005679

[B185] Orvedahl A. Levine B. (2008). Viral evasion of autophagy. Autophagy 4, 280–285. 10.4161/auto.5289 18059171 PMC3508671

[B186] Papassotiropoulos A. Lambert J. C. Wavrant-De Vrièze F. Wollmer M. A. von der Kammer H. Streffer J. R.. (2005). Cholesterol 25-hydroxylase on chromosome 10q is a susceptibility gene for sporadic Alzheimer’s disease. Neurodegener. Dis. 2, 233–241. 10.1159/000090362 16909003

[B187] Pereira F. A. (1996). Herpes simplex: evolving concepts. J. Am. Acad. Dermatol. 35, 503–520; quiz 521–522. 10.1016/s0190-9622(96)90670-2 8859276

[B188] Perng G. C. Jones C. (2010). Towards an understanding of the herpes simplex virus type 1 latency-reactivation cycle. Interdiscip. Perspect. Infect. Dis. 2010:262415. 10.1155/2010/262415 20169002 PMC2822239

[B189] Piacentini R. Civitelli L. Ripoli C. Marcocci M. E. De Chiara G. Garaci E.. (2011). HSV-1 promotes Ca^2+^-mediated APP phosphorylation and Aβ accumulation in rat cortical neurons. Neurobiol. Aging 32, 2323.e13–2323.e26. 10.1016/j.neurobiolaging.2010.06.009 20674092

[B190] Piacentini R. Li Puma D. D. Ripoli C. Marcocci M. E. De Chiara G. Garaci E.. (2015). Herpes simplex virus type-1 infection induces synaptic dysfunction in cultured cortical neurons via GSK-3 activation and intraneuronal amyloid-β protein accumulation. Sci. Rep. 5:15444. 10.1038/srep15444 26487282 PMC4614347

[B191] Pires de Mello C. P. Bloom D. C. Paixão I. C. (2016). Herpes simplex virus type-1: replication, latency, reactivation and its antiviral targets. Antivir Ther. 21, 277–286. 10.3851/IMP3018 26726828

[B192] Pisa D. Alonso R. Juarranz A. Rábano A. Carrasco L. (2015a). Direct visualization of fungal infection in brains from patients with Alzheimer’s disease. J. Alzheimers Dis. 43, 613–624. 10.3233/JAD-141386 25125470

[B193] Pisa D. Alonso R. Rábano A. Rodal I. Carrasco L. (2015b). Different brain regions are infected with fungi in Alzheimer’s disease. Sci. Rep. 5:15015. 10.1038/srep15015 26468932 PMC4606562

[B194] Porcellini E. Carbone I. Ianni M. Licastro F. (2010). Alzheimer’s disease gene signature says: beware of brain viral infections. Immun. Ageing 7:16. 10.1186/1742-4933-7-16 21156047 PMC3019140

[B195] Pouplin T. Pouplin J. N. Van Toi P. Lindegardh N. Rogier van Doorn H. Hien T. T.. (2011). Valacyclovir for herpes simplex encephalitis. Antimicrob. Agents Chemother. 55, 3624–3626. 10.1128/AAC.01023-10 21576427 PMC3122427

[B196] Prasad K. M. Eack S. M. Keshavan M. S. Yolken R. H. Iyengar S. Nimgaonkar V. L. (2013). Antiherpes virus-specific treatment and cognition in schizophrenia: a test-of concept randomized double-blind placebo-controlled trial. Schizophr. Bull. 39, 857–866. 10.1093/schbul/sbs040 22446565 PMC3686443

[B197] Prasad K. M. Shirts B. H. Yolken R. H. Keshavan M. S. Nimgaonkar V. L. (2007). Brain morphological changes associated with exposure to HSV1 in first-episode schizophrenia. Mol. Psychiatry 12, 105–113. 10.1038/sj.mp.4001915 17033628

[B198] Prasad K. M. Watson A. M. Dickerson F. B. Yolken R. H. Nimgaonkar V. L. (2012). Exposure to herpes simplex virus type 1 and cognitive impairments in individuals with schizophrenia. Schizophr. Bull. 38, 1137–1148. 10.1093/schbul/sbs046 22490995 PMC3494052

[B199] Price D. A. Bassendine M. F. Norris S. M. Golding C. Toms G. L. Schmid M. L.. (2006). Apolipoprotein ɛ3 allele is associated with persistent hepatitis C virus infection. Gut 55, 715–718. 10.1136/gut.2005.079905 16299033 PMC1856106

[B200] Prince M. Comas-Herrera A. Knapp M. Guerchet M. Karagiannidou M. (2016). World Alzheimer Report 2016. Improving Healthcare for People Living with Dementia. London: Alzheimer’s Disease International Available online at: https://www.alz.co.uk/research/WorldAlzheimerReport2016.pdf.

[B201] Puzzo D. Vitolo O. Trinchese F. Jacob J. P. Palmeri A. Arancio O. (2005). Amyloid-β peptide inhibits activation of the nitric oxide/cGMP/cAMP-responsive element-binding protein pathway during hippocampal synaptic plasticity. J. Neurosci. 25, 6887–6897. 10.1523/JNEUROSCI.5291-04.2005 16033898 PMC6725343

[B202] Rajmohan R. Reddy P. H. (2017). Amyloid-β and phosphorylated tau accumulations cause abnormalities at synapses of Alzheimer’s disease neurons. J. Alzheimers Dis. 57, 975–999. 10.3233/JAD-160612 27567878 PMC5793225

[B203] Ramakrishna C. Ferraioli A. Calle A. Nguyen T. K. Openshaw H. Lundberg P. S.. (2015). Establishment of HSV1 latency in immunodeficient mice facilitates efficient *in vivo* reactivation. PLoS Pathog. 11:e1004730. 10.1371/journal.ppat.1004730 25760441 PMC4356590

[B204] Reddy P. H. Mani G. Park B. S. Jacques J. Murdoch G. Whetsell W. Jr.. (2005). Differential loss of synaptic proteins in Alzheimer’s disease: implications for synaptic dysfunction. J. Alzheimers Dis. 7, 103–117; discussion 173–180. 10.3233/jad-2005-7203 15851848

[B205] Roberts R. O. Christianson T. J. Kremers W. K. Mielke M. M. Machulda M. M. Vassilaki M.. (2016). Association between olfactory dysfunction and amnestic mild cognitive impairment and Alzheimer disease dementia. JAMA Neurol. 73, 93–101. 10.1001/jamaneurol.2015.2952 26569387 PMC4710557

[B206] Roubaud Baudron C. Letenneur L. Langlais A. Buissonnière A. Mégraud F. Dartigues J. F.. (2013). Does Helicobacter pylori infection increase incidence of dementia? The Personnes Agées QUID Study. J. Am. Geriatr. Soc. 61, 74–78. 10.1111/jgs.12065 23252507

[B207] Saffran H. A. Pare J. M. Corcoran J. A. Weller S. K. Smiley J. R. (2007). Herpes simplex virus eliminates host mitochondrial DNA. EMBO Rep. 8, 188–193. 10.1038/sj.embor.7400878 17186027 PMC1796774

[B208] Sakamoto K. Karelina K. Obrietan K. (2011). CREB: a multifaceted regulator of neuronal plasticity and protection. J. Neurochem. 116, 1–9. 10.1111/j.1471-4159.2010.07080.x 21044077 PMC3575743

[B209] Saldanha J. Sutton R. N. Gannicliffe A. Farragher B. Itzhaki R. F. (1986). Detection of HSV1 DNA by *in situ* hybridisation in human brain after immunosuppression. J. Neurol. Neurosurg. Psychiatry 49, 613–619. 10.1136/jnnp.49.6.613 3016195 PMC1028840

[B210] Santana S. Bullido M. J. Recuero M. Valdivieso F. Aldudo J. (2012a). Herpes simplex virus type I induces an incomplete autophagic response in human neuroblastoma cells. J. Alzheimers Dis. 30, 815–831. 10.3233/JAD-2012-112000 22475795

[B211] Santana S. Recuero M. Bullido M. J. Valdivieso F. Aldudo J. (2012b). Herpes simplex virus type I induces the accumulation of intracellular β-amyloid in autophagic compartments and the inhibition of the non-amyloidogenic pathway in human neuroblastoma cells. Neurobiol. Aging 33, 430.e19–430.e33. 10.1016/j.neurobiolaging.2010.12.010 21272962

[B212] Santana S. Sastre I. Recuero M. Bullido M. J. Aldudo J. (2013). Oxidative stress enhances neurodegeneration markers induced by herpes simplex virus type 1 infection in human neuroblastoma cells. PLoS One 8:e75842. 10.1371/journal.pone.0075842 24124518 PMC3790872

[B213] Satpute-Krishnan P. DeGiorgis J. A. Bearer E. L. (2003). Fast anterograde transport of herpes simplex virus: role for the amyloid precursor protein of Alzheimer’s disease. Aging Cell 2, 305–318. 10.1046/j.1474-9728.2003.00069.x 14677633 PMC3622731

[B215] Sawtell N. M. (1998). The probability of *in vivo* reactivation of herpes simplex virus type 1 increases with the number of latently infected neurons in the ganglia. J. Virol. 72, 6888–6892. 9658140 10.1128/jvi.72.8.6888-6892.1998 PMC109900

[B214] Sawtell N. M. Bernstein D. I. Stanberry L. R. (1999). A temporal analysis of acyclovir inhibition of induced herpes simplex virus type 1 *in vivo* reactivation in the mouse trigeminal ganglia. J. Infect. Dis. 180, 821–823. 10.1086/314958 10438371

[B216] Schachtele S. J. Hu S. Little M. R. Lokensgard J. R. (2010). Herpes simplex virus induces neural oxidative damage via microglial cell Toll-like receptor-2. J. Neuroinflammation 7:35. 10.1186/1742-2094-7-35 20584314 PMC2904293

[B217] Scheff S. W. Ansari M. A. Mufson E. J. (2016). Oxidative stress and hippocampal synaptic protein levels in elderly cognitively intact individuals with Alzheimer’s disease pathology. Neurobiol. Aging 42, 1–12. 10.1016/j.neurobiolaging.2016.02.030 27143416 PMC4857887

[B218] Schretlen D. J. Vannorsdall T. D. Winicki J. M. Mushtaq Y. Hikida T. Sawa A.. (2010). Neuroanatomic and cognitive abnormalities related to herpes simplex virus type 1 in schizophrenia. Schizophr. Res. 118, 224–231. 10.1016/j.schres.2010.01.008 20153952

[B219] Schubert C. R. Carmichael L. L. Murphy C. Klein B. E. Klein R. Cruickshanks K. J. (2008). Olfaction and the 5-year incidence of cognitive impairment in an epidemiological study of older adults. J. Am. Geriatr. Soc. 56, 1517–1521. 10.1111/j.1532-5415.2008.01826.x 18662205 PMC2587240

[B220] Shah N. H. Aizenman E. (2014). Voltage-gated potassium channels at the crossroads of neuronal function, ischemic tolerance, and neurodegeneration. Transl Stroke Res. 5, 38–58. 10.1007/s12975-013-0297-7 24323720 PMC3946373

[B221] Shen J. H. Huang K. Y. Chao-Yu C. Chen C. J. Lin T. Y. Huang Y. C. (2015). Seroprevalence of herpes simplex virus type 1 and 2 in taiwan and risk factor analysis, 2007. PLoS One 10:e0134178. 10.1371/journal.pone.0134178 26252011 PMC4529201

[B222] Shimeld C. Whiteland J. L. Nicholls S. M. Grinfeld E. Easty D. L. Gao H.. (1995). Immune cell infiltration and persistence in the mouse trigeminal ganglion after infection of the cornea with herpes simplex virus type 1. J. Neuroimmunol. 61, 7–16. 10.1016/0165-5728(95)00068-d 7560014

[B223] Shimeld C. Whiteland J. L. Williams N. A. Easty D. L. Hill T. J. (1997). Cytokine production in the nervous system of mice during acute and latent infection with herpes simplex virus type 1. J. Gen. Virol. 78, 3317–3325. 10.1099/0022-1317-78-12-3317 9400983

[B224] Shipley S. J. Parkin E. T. Itzhaki R. F. Dobson C. B. (2005). Herpes simplex virus interferes with amyloid precursor protein processing. BMC Microbiol. 5:48. 10.1186/1471-2180-5-48 16109164 PMC1198230

[B225] Shirts B. H. Prasad K. M. Pogue-Geile M. F. Dickerson F. Yolken R. H. Nimgaonkar V. L. (2008). Antibodies to cytomegalovirus and herpes simplex virus 1 associated with cognitive function in schizophrenia. Schizophr. Res. 106, 268–274. 10.1016/j.schres.2008.07.017 18801645 PMC2615667

[B226] Shives K. D. Tyler K. L. Beckham J. D. (2017). Molecular mechanisms of neuroinflammation and injury during acute viral encephalitis. J. Neuroimmunol. 308, 102–111. 10.1016/j.jneuroim.2017.03.006 28291542 PMC12101058

[B228] Smith J. S. Robinson N. J. (2002). Age-specific prevalence of infection with herpes simplex virus types 2 and 1: a global review. J. Infect. Dis. 186, S3–S28. 10.1086/343739 12353183

[B229] Smith J. D. de Harven E. (1978). Herpes simplex virus and human cytomegalovirus replication in WI-38 cells. J. Virol. 26, 102–109. 206717 10.1128/jvi.26.1.102-109.1978 PMC354038

[B227] Smith J. P. Weller S. Johnson B. Nicotera J. Luther J. M. Haas D. W. (2010). Pharmacokinetics of acyclovir and its metabolites in cerebrospinal fluid and systemic circulation after administration of high-dose valacyclovir in subjects with normal and impaired renal function. Antimicrob. Agents Chemother. 54, 1146–1151. 10.1128/AAC.00729-09 20038622 PMC2825963

[B230] Soontornniyomkij V. Moore D. J. Gouaux B. Soontornniyomkij B. Tatro E. T. Umlauf A.. (2012). Cerebral β-amyloid deposition predicts HIV-associated neurocognitive disorders in APOE ε4 carriers. AIDS 26, 2327–2335. 10.1097/QAD.0b013e32835a117c 23018443 PMC3576852

[B231] Soscia S. J. Kirby J. E. Washicosky K. J. Tucker S. M. Ingelsson M. Hyman B.. (2010). The Alzheimer’s disease-associated amyloid β-protein is an antimicrobial peptide. PLoS One 5:e9505. 10.1371/journal.pone.0009505 20209079 PMC2831066

[B232] Spear P. G. (2004). Herpes simplex virus: receptors and ligands for cell entry. Cell. Microbiol. 6, 401–410. 10.1111/j.1462-5822.2004.00389.x 15056211

[B233] Spitzer P. Condic M. Herrmann M. Oberstein T. J. Scharin-Mehlmann M. Gilbert D. F.. (2016). Amyloidogenic amyloid-β-peptide variants induce microbial agglutination and exert antimicrobial activity. Sci. Rep. 6:32228. 10.1038/srep32228 27624303 PMC5021948

[B234] St. Leger A. J. Hendricks R. L. (2011). CD8+ T cells patrol HSV-1-infected trigeminal ganglia and prevent viral reactivation. J. Neurovirol. 17, 528–534. 10.1007/s13365-011-0062-1 22161682

[B235] Stanga S. Lanni C. Govoni S. Uberti D. D’Orazi G. Racchi M. (2010). Unfolded p53 in the pathogenesis of Alzheimer’s disease: is HIPK2 the link? Aging (Albany, NY) 2, 545–554. 10.18632/aging.100205 20876941 PMC2984604

[B236] Steel A. J. Eslick G. D. (2015). Herpes viruses increase the risk of Alzheimer’s disease: a meta-analysis. J. Alzheimers Dis. 47, 351–364. 10.3233/JAD-140822 26401558

[B237] Stowe R. P. Kozlova E. V. Yetman D. L. Walling D. M. Goodwin J. S. Glaser R. (2007). Chronic herpesvirus reactivation occurs in aging. Exp. Gerontol. 42, 563–570. 10.1016/j.exger.2007.01.005 17337145 PMC1992441

[B238] Stowe R. P. Peek M. K. Cutchin M. P. Goodwin J. S. (2012). Reactivation of herpes simplex virus type 1 is associated with cytomegalovirus and age. J. Med. Virol. 84, 1797–1802. 10.1002/jmv.23397 22997083 PMC3463941

[B240] Strandberg T. E. Aiello A. E. (2013). Is the microbe-dementia hypothesis finally ready for a treatment trial? Neurology 80, 1182–1183. 10.1212/WNL.0b013e3182897126 23530150

[B239] Strandberg T. Pitkala K. Linnavuori K. Tilvis R. (2003). Impact of viral and bacterial burden on cognitive impairment in elderly persons with cardiovascular disease. Stroke 34, 2126–2131. 10.1161/01.str.0000086754.32238.da 12920256

[B241] Takahashi R. H. Nagao T. Gouras G. K. (2017). Plaque formation and the intraneuronal accumulation of β-amyloid in Alzheimer’s disease. Pathol. Int. 67, 185–193. 10.1111/pin.12520 28261941

[B242] Tallóczy Z. Jiang W. Virgin H. W. IV. Leib D. A. Scheuner D. Kaufman R. J.. (2002). Regulation of starvation- and virus-induced autophagy by the eIF2α kinase signaling pathway. Proc. Natl. Acad. Sci. U S A 99, 190–195. 10.1073/pnas.012485299 11756670 PMC117537

[B243] Tallóczy Z. Virgin H. W. IV. Levine B. (2006). PKR-dependent autophagic degradation of herpes simplex virus type 1. Autophagy 2, 24–29. 10.4161/auto.2176 16874088

[B244] Tarter K. D. Simanek A. M. Dowd J. B. Aiello A. E. (2014). Persistent viral pathogens and cognitive impairment across the life course in the third national health and nutrition examination survey. J. Infect. Dis. 209, 837–844. 10.1093/infdis/jit616 24253286 PMC3935478

[B245] Teich A. F. Nicholls R. E. Puzzo D. Fiorito J. Purgatorio R. Fa’ M.. (2015). Synaptic therapy in Alzheimer’s disease: a CREB-centric approach. Neurotherapeutics 12, 29–41. 10.1007/s13311-014-0327-5 25575647 PMC4322064

[B246] Thomas R. J. (1995). Excitatory amino acids in health and disease. J. Am. Geriatr. Soc. 43, 1279–1289. 10.1111/j.1532-5415.1995.tb07407.x 7594165

[B247] Tong L. Thornton P. L. Balazs R. Cotman C. W. (2001). β-amyloid-(1–42) impairs activity-dependent cAMP-response element-binding protein signaling in neurons at concentrations in which cell survival Is not compromised. J. Biol. Chem. 276, 17301–17306. 10.1007/978-3-319-67199-4_100522 11278679

[B248] Tönnies E. Trushina E. (2017). Oxidative stress, synaptic dysfunction and Alzheimer’s disease. J. Alzheimers Dis. 57, 1105–1121. 10.3233/JAD-161088 28059794 PMC5409043

[B249] Torrent M. Pulido D. Nogués M. V. Boix E. (2012). Exploring new biological functions of amyloids: bacteria cell agglutination mediated by host protein aggregation. PLoS Pathog. 8:e1003005. 10.1371/journal.ppat.1003005 23133388 PMC3486885

[B250] Trushina E. Nemutlu E. Zhang S. Christensen T. Camp J. Mesa J.. (2012). Defects in mitochondrial dynamics and metabolomic signatures of evolving energetic stress in mouse models of familial Alzheimer’s disease. PLoS One 7:e32737. 10.1371/journal.pone.0032737 22393443 PMC3290628

[B251] Tyring S. K. Baker D. Snowden W. (2002). Valacyclovir for herpes simplex virus infection: long-term safety and sustained efficacy after 20 years′ experience with acyclovir. J. Infect. Dis. 186, S40–S46. 10.1086/342966 12353186

[B252] Valyi-Nagy T. Dermody T. S. (2005). Role of oxidative damage in the pathogenesis of viral infections of the nervous system. Histol. Histopathol. 20, 957–967. 10.14670/HH-20.957 15944946

[B253] Veerhuis R. Janssen I. Hack C. E. Eikelenboom P. (1996). Early complement components in Alzheimer’s disease brains. Acta Neuropathol. 91, 53–60. 10.1007/s004019570001 8773146

[B255] Vitolo O. V. Sant’Angelo A. Costanzo V. Battaglia F. Arancio O. Shelanski M. (2002). Amyloid β-peptide inhibition of the PKA/CREB pathway and long-term potentiation: reversibility by drugs that enhance cAMP signaling. Proc. Natl. Acad. Sci. U S A 99, 13217–13221. 10.1073/pnas.172504199 12244210 PMC130613

[B256] Wang X. Su B. Siedlak S. L. Moreira P. I. Fujioka H. Wang Y.. (2008). Amyloid-β overproduction causes abnormal mitochondrial dynamics via differential modulation of mitochondrial fission/fusion proteins. Proc. Natl. Acad. Sci. U S A 105, 19318–19323. 10.1073/pnas.0804871105 19050078 PMC2614759

[B257] Wang X. Su B. Zheng L. Perry G. Smith M. A. Zhu X. (2009). The role of abnormal mitochondrial dynamics in the pathogenesis of Alzheimer’s disease. J. Neurochem. 109, 153–159. 10.1111/j.1471-4159.2009.05867.x 19393022 PMC2720030

[B258] Wang X. Wang W. Li L. Perry G. Lee H. G. Zhu X. (2014). Oxidative stress and mitochondrial dysfunction in Alzheimer’s disease. Biochim. Biophys. Acta 1842, 1240–1247. 10.1016/j.bbadis.2013.10.015 24189435 PMC4007397

[B259] Wang X. L. Zeng J. Feng J. Tian Y. T. Liu Y. J. Qiu M.. (2014). Helicobacter pylori filtrate impairs spatial learning and memory in rats and increases β-amyloid by enhancing expression of presenilin-2. Front. Aging Neurosci. 6:66. 10.3389/fnagi.2014.00066 24782763 PMC3990046

[B260] White M. R. Kandel R. Tripathi S. Condon D. Qi L. Taubenberger J.. (2014). Alzheimer’s associated β-amyloid protein inhibits influenza A virus and modulates viral interactions with phagocytes. PLoS One 9:e101364. 10.1371/journal.pone.0101364 24988208 PMC4079246

[B261] Wilcox D. R. Longnecker R. (2016). The herpes simplex virus neurovirulence factor γ34.5: revealing virus-host interactions. PLoS Pathog. 12:e1005449. 10.1371/journal.ppat.1005449 26964062 PMC4786305

[B262] Williams W. M. Torres S. Siedlak S. L. Castellani R. J. Perry G. Smith M. A.. (2013). Antimicrobial peptide β-defensin-1 expression is upregulated in Alzheimer’s brain. J. Neuroinflammation 10:127. 10.1186/1742-2094-10-127 24139179 PMC3817866

[B263] Woodward M. R. Amrutkar C. V. Shah H. C. Benedict R. H. Rajakrishnan S. Doody R. S.. (2017). Validation of olfactory deficit as a biomarker of Alzheimer disease. Neurol. Clin. Pract. 7, 5–14. 10.1212/CPJ.0000000000000293 28243501 PMC5310210

[B264] World Health Organization (2017). Herpes simplex virus. Available online at: http://www.who.int/mediacentre/factsheets/fs400/en [Accessed on January 27, 2018].

[B265] Wozniak M. A. Frost A. L. Itzhaki R. F. (2009a). Alzheimer’s disease-specific tau phosphorylation is induced by herpes simplex virus type 1. J. Alzheimers Dis. 16, 341–350. 10.3233/JAD-2009-0963 19221424

[B269] Wozniak M. A. Mee A. P. Itzhaki R. F. (2009b). Herpes simplex virus type 1 DNA is located within Alzheimer’s disease amyloid plaques. J. Pathol. 217, 131–138. 10.1002/path.2449 18973185

[B266] Wozniak M. A. Frost A. L. Preston C. M. Itzhaki R. F. (2011). Antivirals reduce the formation of key Alzheimer’s disease molecules in cell cultures acutely infected with Herpes simplex virus type 1. PLoS One 6:e25152. 10.1371/journal.pone.0025152 22003387 PMC3189195

[B267] Wozniak M. A. Itzhaki R. F. Faragher E. B. James M. W. Ryder S. D. Irving W. L. (2002). Apolipoprotein E-ɛ 4 protects against severe liver disease caused by hepatitis C virus. Hepatology 36, 456–463. 10.1053/jhep.2002.34745 12143056

[B268] Wozniak M. A. Itzhaki R. F. Shipley S. J. Dobson C. B. (2007). Herpes simplex virus infection causes cellular amyloid accumulation and secretase up-regulation. Neurosci. Lett. 429, 95–100. 10.1016/j.neulet.2007.09.077 17980964

[B270] Wozniak M. A. Shipley S. J. Combrinck M. Wilcock G. K. Itzhaki R. F. (2005). Productive herpes virus in brain of elderly normal subjects and Alzheimer’s disease patients. J. Med. Virol. 75, 300–306. 10.1002/jmv.20271 15602731

[B271] Wyss-Coray T. Rogers J. (2012). Inflammation in Alzheimer disease-a brief review of the basic science and clinical literature. Cold Spring Harb. Perspect. Med. 2:a006346. 10.1101/cshperspect.a006346 22315714 PMC3253025

[B272] Xia M. Q. Hyman B. T. (1999). Chemokines/chemokine receptors in the central nervous system and Alzheimer’s disease. J. Neurovirol. 5, 32–41. 10.3109/13550289909029743 10190688

[B273] Yamamoto-Sasaki M. Ozawa H. Saito T. Rösler M. Riederer P. (1999). Impaired phosphorylation of cyclic AMP response element binding protein in the hippocampus of dementia of the Alzheimer type. Brain Res. 824, 300–303. 10.1016/s0006-8993(99)01220-2 10196463

[B274] Yao H. W. Ling P. Tung Y. Y. Hsu S. M. Chen S. H. (2014). *In vivo* reactivation of latent herpes simplex virus 1 in mice can occur in the brain before occurring in the trigeminal ganglion. J. Virol. 88, 11264–11270. 10.1128/JVI.01616-14 25031345 PMC4178801

[B275] Yasojima K. Akiyama H. McGeer E. G. McGeer P. L. (2001). Reduced neprilysin in high plaque areas of Alzheimer brain: a possible relationship to deficient degradation of β-amyloid peptide. Neurosci. Lett. 297, 97–100. 10.1016/s0304-3940(00)01675-x 11121879

[B276] Yolken R. H. Torrey E. F. Lieberman J. A. Yang S. Dickerson F. B. (2011). Serological evidence of exposure to herpes simplex virus type 1 is associated with cognitive deficits in the CATIE schizophrenia sample. Schizophr. Res. 128, 61–65. 10.1016/j.schres.2011.01.020 21353483

[B277] Yu X. He S. (2016). The interplay between human herpes simplex virus infection and the apoptosis and necroptosis cell death pathways. Virol. J. 13:77. 10.1186/s12985-016-0528-0 27154074 PMC4859980

[B278] Zambrano A. Solis L. Salvadores N. Cortés M. Lerchundi R. Otth C. (2008). Neuronal cytoskeletal dynamic modification and neurodegeneration induced by infection with herpes simplex virus type 1. J. Alzheimers Dis. 14, 259–269. 10.3233/jad-2008-14301 18599953

[B279] Zhao Y. Zhao B. (2013). Oxidative stress and the pathogenesis of Alzheimer’s disease. Oxid. Med. Cell. Longev. 2013:316523. 10.1155/2013/316523 23983897 PMC3745981

